# Developing the PEAK mood, mind, and marks program to support university students’ mental and cognitive health through physical exercise: a qualitative study using the Behaviour Change Wheel

**DOI:** 10.1186/s12889-024-19385-x

**Published:** 2024-07-23

**Authors:** Catherine E. B. Brown, Karyn Richardson, Bengianni Halil-Pizzirani, Sam Hughes, Lou Atkins, Rachel Perowne, Joseph Pitt, Murat Yücel, Rebecca A. Segrave

**Affiliations:** 1 Turner Institute for Brain and Mental Health, Monash University, Melbourne, VIC Australia; 2 Centre for Behaviour Change, University College London, London, UK; 3 QIMR Berghofer Medical Research Institute, Herston, Brisbane, QLD Australia

**Keywords:** University students, Co-design, Physical exercise, Mental health, Cognitive health, Behavioural intervention, Behaviour change wheel, COM-B

## Abstract

**Background:**

Concerns about mental and cognitive health are common among university students. Engaging in regular physical exercise has been shown to enhance both mental health and cognitive performance, yet most students are not participating in the level of exercise required to obtain these benefits. The Behaviour Change Wheel (BCW) provides a framework for developing behavioural interventions that are informed by theory, evidence, and stakeholder perspectives. The current study aimed to apply the BCW to develop the PEAK Mood, Mind, and Marks program (i.e., PEAK), a behaviour change intervention designed to increase university students’ exercise engagement for the benefit of their mental and cognitive health.

**Methods:**

PEAK was developed across three stages of the BCW: (1) understand the target behaviour, (2) identify intervention options, and (3) identify intervention content and delivery mode. Development was informed by triangulated data from a systematic literature review, co-design consultations with key stakeholders, and knowledge of relevant experts. Consultations with stakeholders involved focus groups with 25 university students and individual interviews with 10 university leaders and staff to identify barriers and facilitators to students’ exercise engagement and the adoption and implementation of PEAK by universities. Template analysis was used to code transcripts to the capability, opportunity, and motivation (COM-B) model of behaviour. The BCW was applied to identify the most appropriate intervention types and behaviour change techniques (BCTs).

**Results:**

Thirty-one barriers and facilitators were identified and mapped to seven intervention types (Education; Modelling; Persuasion; Environmental Restructuring; Incentivisation; Training; and Enablement) and 26 BCTs, which were delivered across digital channels and in-person. The final intervention consisted of multiple components targeting students’ capability (e.g., increasing knowledge about the mental and cognitive health benefits of exercise), opportunity (e.g., providing a flexible range of accessible exercise options and social support), and motivation (e.g., increasing the perceived importance of exercise) to exercise.

**Conclusions:**

University students and staff describe a need and appetite for more empowering, scalable solutions to support students’ mental and cognitive health. Exercise-based approaches that are informed by behaviour change frameworks, evidence, and stakeholder perspectives, such as PEAK, have the potential to address this need. Current findings will inform a pilot of PEAK to evaluate its efficacy and implementation.

**Supplementary Information:**

The online version contains supplementary material available at 10.1186/s12889-024-19385-x.

## Background

The mental and cognitive health of university students is of growing concern for the global tertiary education sector. A multinational study of approximately 14,000 university students estimated that 35% met the criteria for at least one anxiety, mood, or substance disorder [1]. Poor mental health is often associated with cognitive difficulties in areas such as memory, attention and processing speed [2, 3]. Cognitive concerns are common even among students who do not meet the criteria for a mental illness. For instance, an Australian survey of 901 university students found that more than 60% were concerned about their cognition, including their ability to recall memories and concentrate, irrespective of their mental health status [4]. Given that mental and cognitive health are important determinants of academic success, degree completion and future career prospects [5–7], there is a pressing need for effective strategies to address these issues.

Promoting university students’ engagement in physical exercise has the potential to enhance their mental health and cognitive performance. There is robust evidence that regular exercise can reduce the severity of anxiety and depression [8] and, when performed at moderate to high intensities, can have comparable therapeutic effects to psychotherapy and pharmacotherapy [9]. Even three exercise sessions per week has been associated with significant reductions in days of poor mental health [10]. Importantly, the same exercise prescriptions that benefit mental health also benefit cognition. Regular exercise can increase cognitive performance across numerous domains including executive functions [11], attention [12] and memory [13]. The mental and cognitive health benefits of exercise are partly attributed to exercise-induced neuroplasticity, including alterations in neurotrophin availability and brain volume, connectivity, and functioning, especially in regions such as the hippocampus that are involved in learning, memory, and emotional processing [14–16]. The benefits of exercise are well-established; however, most university students are not currently engaging in the frequency or intensity of exercise that best supports optimal mental and cognitive health. Globally, only 13–32% of university students exercise three or more times a week [17]. Low rates of exercise among students are largely attributed to the unique challenges that often accompany the transition to university life, such as new academic pressures, social adjustments, and financial responsibilities [18], which disrupt established routines and pose additional barriers to the initiation or maintenance of regular exercise [19].

Although initiating or maintaining health behaviours is challenging, many of the key principles for developing successful behaviour change programs are well established. The UK Medical Research Council (MRC) guidelines for developing complex interventions [20] and National Institute for Health and Care Excellence (NICE) [21–23] advocates an evidence-based approach that incorporates stakeholder perspectives at all stages of the intervention development process. Involving key stakeholders (in the context of this research, university students, leadership, and staff) in intervention development is important to maximise the feasibility, adoption, and scalability potential of an intervention within the university setting [24]. Effective behaviour change interventions are most often created when stakeholder perspectives are incorporated with proven evidence-based frameworks from behavioural science [25, 26].

**Fig. 1:**
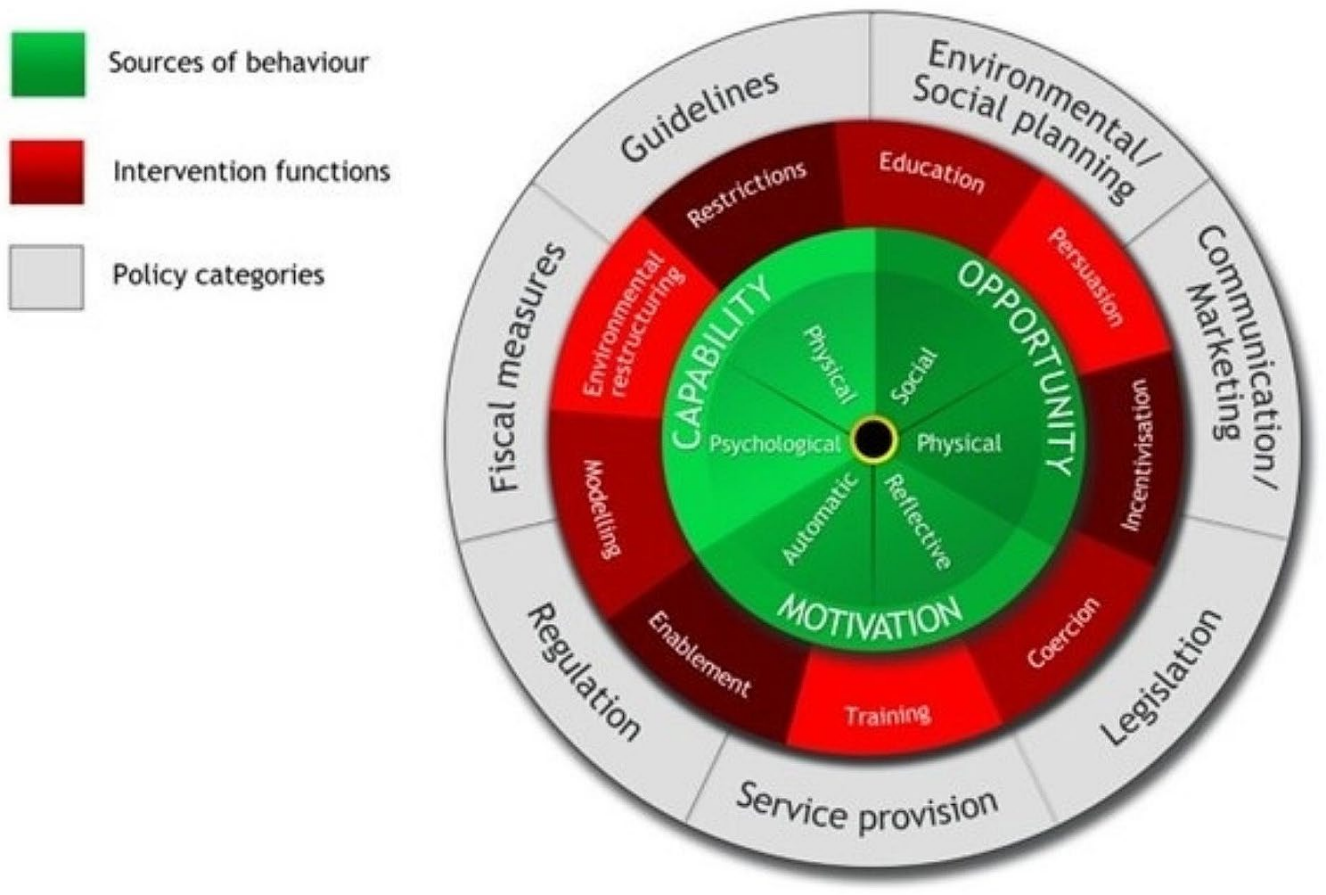
The three layers of the behaviour change wheel. reproduced from [31]

The Behaviour Change Wheel (BCW) is globally recognised as one of the most well validated, theoretically derived, frameworks for developing and describing behaviour change interventions [27]. The BCW has been widely used by clinicians, governments, industry and non-governmental associations around the world to create large scale change across healthcare, environmental sustainability, and workplace settings [28–30]. The BCW is built on a synthesis of 19 behaviour change frameworks schematically organised into three layers (see Fig. 1) which form the basis of a systematic process for intervention development across three stages: (1) understanding the target behaviour, (2) identifying intervention options, and (3) identifying intervention content and delivery mode [31]. The theoretical framework used to understand the behaviour is the COM-B model, which posits that no behaviour will occur without sufficient capability, opportunity, and motivation. Within the COM-B model, capability can be psychological (e.g., knowledge to engage in the necessary processes) or physical (e.g., physical skills); opportunity can be social (e.g., interpersonal influences) or physical (e.g., environmental resources); and motivation can be automatic (e.g., emotional reactions, habits) or reflective (e.g., intentions, beliefs). This model highlights that where any of these are lacking, they can be strategically targeted to increase the likelihood of a person changing their behaviour.

The second stage of intervention development involves selecting from nine intervention types that were mapped onto the COM-B model through expert consensus (see Additional file 1 for definitions and examples of each intervention type). Through this process, behavioural scientists reached an agreement on which intervention types were most likely to change behaviour by targeting capability, opportunity and/or motivation as required. The intervention types are also linked to seven general policy options that can be used to support the delivery of the intervention types when relevant and/or available to intervention designers.

The final stage of the intervention development process includes identifying which behaviour change techniques (BCTs) can be used to support the delivery of intervention types and appropriate modes of delivery. BCTs are defined as the observable and replicable ‘active ingredients’ of behaviour change interventions that influence behaviour as described in a BCT taxonomy (BCTTv1) [32]. The taxonomy was developed through a Delphi process to provide an extensive, consensually agreed, and standardised categorisation of 93 BCTs used in behaviour change interventions.

The BCW provides a rigorous theoretically derived framework to guide the development of a behavioural intervention aiming to support university students’ engagement in exercise to improve their mental and cognitive health. As the BCW process has not yet been applied to design an intervention of this kind, the current study aimed to apply the BCW to develop the PEAK Mood, Mind, and Marks program (i.e., PEAK)^1^, a behaviour change intervention designed to increase university students’ exercise for the benefit of their mental and cognitive health.

## Methods

**Fig. 2:**
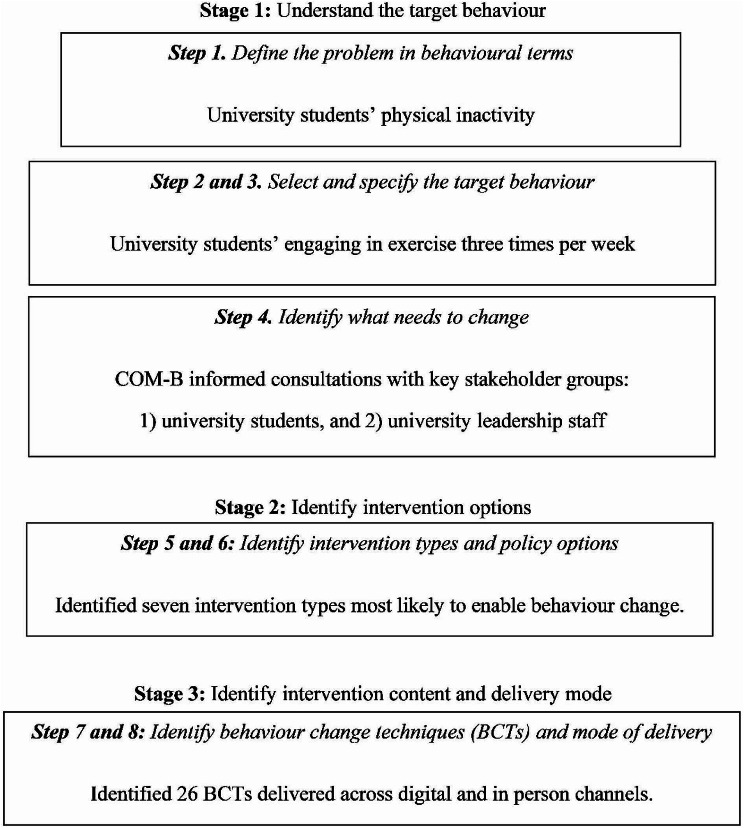
The eight steps of the behaviour change wheel applied to develop the PEAK mood, mind, and marks program

This study utilised a qualitative study design, guided by the BCW process and person-based approach to intervention development [27, 33]. The BCW process outlines eight steps across the three stages of the intervention development process, each of which was followed to develop PEAK (see Fig. 2). An iterative feedback loop approach was taken, wherein findings from each step fed into the next step of the development process and were also fed back to refine previous steps. Results for steps one through three have been described in the methods below to provide contextual information for the qualitative research methods described in step four. This study was approved by the Monash University Human Research Ethics Committee (Reference Number 27730).

### Stage 1: understand the target behaviour

#### Step 1: define the problem in behavioural terms

The first step was to identify the problem behaviour that the intervention needed to address (e.g., physical inactivity). This included specifying: (1) who was performing the behaviour, and (2) what the behaviour involved. Most students are currently not engaging in the frequency or intensity of exercise that best supports peak mental and cognitive health. Interventions that support students to engage in regular exercise will therefore be beneficial.

#### Step 2 and step 3: select and specify the target behaviour

The target behaviour was for students to build up to engaging in three or more exercise sessions per week. This frequency was informed by evidence that suggests exercising three times per week may generate the largest improvements in mental health [10]. A session was defined as purposeful and effortful exercise that lasted for 10-minutes or longer. This definition is in keeping with the International Physical Activity Questionnaire, an internationally recognised, widely used, and validated measure of physical activity [34]. The behaviour was specified with regards to who needed to perform the target behaviour, what they needed to do differently to achieve the desired change, when, where, how often they needed to do it and with whom (Table 1).

**Table 1:** Specification of the target behaviour

Target behaviour	Engage in exercise that lasts for 10-minutes or longer three or more times per week
Who needs to perform the behaviour?	Undergraduate university students at Monash University
What do they need to do differently to achieve the desired change?	Engage in exercise that lasts for 10-minutes or longer three or more times per week
When do they need to do it?	Any day throughout a 12-week university teaching semester
Where do they need to do it?	Any environment conducive for exercise (e.g., at home, on-campus, at the gym, etc.)
How often do they need to do it?	Three or more times per week
With whom do they need to do it?	Alone or with others

*Note* Adapted from [27]

#### Step 4: identify what needs to change

Triangulating data from multiple sources is recommended to obtain a deep understanding of what needs to change for the target behaviour to occur. Data was triangulated from three sources:

- *Systematic review.* The research team conducted a systematic review of peer-reviewed research on university students’ barriers and facilitators to exercise [35]. This synthesis was couched within the COM-B model.
- *Key stakeholder perspectives.* An empirical study using a qualitative co-design approach was conducted with university students, senior leadership, and staff at Monash University to explore barriers and facilitators to students’ engagement in exercise and identify program messaging, content, and delivery methods that would support the acceptability, feasibility, adoption, and scalability of PEAK.
- *Expert knowledge.* The expertise of the intervention design team contributed to understanding the target behaviour and design of program content. The team consisted of experts in behavioural science and implementation science (CB, BS, KR, BP, and LA), psychologists (CB, BS, and KR), a senior exercise physiologist (SH), a co-author of The Behaviour Change Wheel: A Guide to Designing Interventions (LA), and two undergraduate university student ambassadors (see acknowledgements).

Triangulated data was mapped to the COM-B categories to identify what barriers and facilitators in relation to students’ capability, opportunity and/or motivation needed to be targeted to maximise the likelihood of behaviour change. The BCW refers to this process as the behavioural diagnosis.

### Key stakeholder perspectives: participants

#### University students

Twenty-five undergraduate university students (*M* age = 21.28, *SD* = 2.32 years; 17 women, eight men) from Monash University participated in this study. The study was advertised via flyers on-campus and social media posts. Stratified random sampling was used to recruit students based on their typical weekly exercise engagement, and whether they were domestic or international students (Table 2), ensuring the sample was representative of the university population and offered a diversity of perspectives. All students provided informed consent and were reimbursed a $50AUD gift voucher.

#### University senior leadership and staff

Ten senior leaders and staff at Monash University (four women, six men) participated in this study. Stratified purposeful sampling was employed to ensure a range of perspectives were captured across different levels of seniority. Staff varied across disciplines and included representatives from the university chief-suite, school leadership and international student engagement team members, university exercise facilities, student mental health support facilities, student residential services, student association groups, and unit co-ordinators (Table 2). These staff members were purposefully invited to participate because their support of the program and institutional knowledge is important for the widespread adoption and scale of the PEAK program. Invitations to participate were disseminated via email. All staff provided informed consent to participate in the study.

**Table 2:** Participant characteristics

Demographic (%)	Frequency (%)
*Gender*	
Woman	17(68)
Man	8(32)
*Typical exercise engagement per week*	
0	8(32)
1–2 sessions	3(12)
3 + sessions	14(56)
*Enrolment status*	
Domestic	16(64)
International	9(36)
*Faculty enrolment*	
Arts	3(12)
Business and Economics	4(16)
Education	3(12)
Engineering	2(8)
Information Technology	2(8)
Law	1(4)
Medicine, Nursing and Health Sciences	6(24)
Science	4(16)
**University Senior Leadership and Staff**	
*Gender*	
Woman	4(40)
Man	6(60)
*Leadership and Staff Roles*	
Chief-suite	1(10)
School leadership team	1(10)
International student engagement team	1(10)
Exercise facilities	2(20)
Student mental health support facilities	1(10)
Student residential services	2(20)
Student association president	1(10)
Unit co-ordinators	1(10)

### Key stakeholder perspectives: data collection

#### University students

Four focus groups were conducted with undergraduate students, lasting for ~ 2.5 h duration and ranging in size from four to seven participants. Groups were held in-person at the university campus, co-facilitated by two authors (CB and RS or KR or BP), and audio-recorded in full. At the beginning of each focus group, students were shown a video “*Exercising smart: how being active can help you perform better in your studies*” (view the video online at https://vimeo.com/ymcavictoria/download/535721809/3ed49f4683), which aimed to communicate the mental, cognitive, and brain health benefits of exercise. This video was created by young adults to address issues that affect young adults as part of the Virtual Y co-design series [36], a joint initiative between the YMCA and VicHealth. The video was shown to facilitate discussions about students’ existing knowledge (or lack thereof) of how exercise impacts mental, cognitive and brain health, and whether learning this information influenced their motivation to exercise.

The interview schedule was guided by the COM-B model and aimed to facilitate discussion on: (1) the perceived need for mental and cognitive health support, (2) appetite for an exercise-based program to provide this support, (3) barriers and facilitators to exercise, 3) acceptable, accessible delivery channels, and (4) strategies to increase the adoption and acceptability of PEAK (see Additional file 2 for the focus group interview schedule).

Students’ demographic data regarding age, gender, exercise history, enrolment faculty and status was collected online using the Qualtrics platform [37] before they participated in the study.

#### Senior leadership and staff

Individual semi-structured interviews were conducted with staff, lasting for ~ 1 h duration. Interviews were conducted online via Zoom platform [38], individually facilitated by authors (CB or RS or KR), and audio-recorded in full for subsequent anonymised transcription.

The interview schedule was guided by the COM-B model and aimed to facilitate discussion on: (1) the need for PEAK, (2) anticipated barriers and facilitators to the implementation, adoption and scale of PEAK, and (3) the capacity for staff to provide resource support for PEAK (see Additional file 3 for the semi-structured interview schedule).

### Key stakeholder perspectives: data analysis

Focus group and interview audio recordings were anonymously transcribed verbatim. Transcripts were analysed using template analysis [39] —a thematic analytic method—which permits a combination of both a priori themes relevant to the research question (e.g., barriers and facilitators to the implementation of PEAK) alongside inductive coding to develop additional emergent themes. A priori themes were guided by the six categories of the COM-B model. While template analysis was conducted with a psychological lens, the eclectic research expertise of the authors provided an opportunity for input from a wider range of perspectives and disciplines (e.g., behavioural science, neuropsychology, clinical psychology, implementation science and evaluative synthesis).

Preliminary coding was conducted independently by two authors (CB and BP or RP) using a priori themes to identify which COM-B categories were relevant to design and/or implement PEAK. Codes were clustered into meaningful groups in which hierarchical and lateral relationships between themes were defined. The authors met to discuss the coding of the two transcripts (designed to increase calibration between authors) and establish an initial template for coding the remaining transcripts. The initial template was further refined through systematic coding of the remaining transcripts conducted by two authors (CB and BP or RP). The final template was re-applied to the full dataset by two authors (CB and BP or RP).

### Stage 2: identify intervention options

#### Step 5 and 6: identify intervention types and policy options

The intervention types most likely to affect behaviour change were selected based on the COM-B behavioural diagnosis conducted in Step 4. The relevant intervention types were assessed using the APEASE criteria: (1) Affordability, (2) Practicability, (3) Effectiveness and cost-effectiveness, (4) Acceptability, (5) Side-effects/safety, and (6) Equity (see Additional file 4 for definitions of each criterion). The APEASE criteria was developed as part of the BCW process to enable intervention designers to select feasible intervention types before trialling an intervention. In the context of developing an intervention to increase students’ engagement in exercise, intervention types were selected on the basis they satisfied the following criteria:

1. Affordability and Practicability: The intervention type could affordably and practicably be delivered to and accessed by university students within the time, budget, and resources available.
2. Effectiveness and cost-effectiveness: The intervention type had the potential to effectively increase students’ engagement in exercise within a real-world context and could be delivered at minimal cost.
3. Acceptability: University students and staff deemed the intervention type acceptable and appropriate.
4. Side-effects/safety: There were no adverse side-effects associated with the intervention type.
5. Equity: The intervention type had the potential to reduce health and wellbeing disparities between various student groups, such as domestic and international students, and women and men.

As policy levers were not deemed to be relevant at this stage of development, identifying policy options as stipulated in step 6 of the BCW process was not undertaken.

### Stage 3: identify intervention content and delivery mode

#### Step 7 and 8: identify behaviour change techniques and mode of delivery

The BCW process describes links between intervention types and individual BCTs. From the list of 93 BCTs identified in the BCTTv1, the most appropriate techniques were selected for the program to facilitate the desired behaviour change. The content and mode of delivery for each BCT was further operationalised to create the overall PEAK program. These decisions were informed by iterative discussions between the intervention design team, including regular consultations with student ambassadors, and application of the APEASE criteria. A 12-week pilot was then planned to run coincident with a full university teaching semester.

## Results

### Stage 1: understanding the problem

Steps one through to three have been described in the methods above. This research has generated new findings from steps four through to eight, which are described below.

#### Step 4: identify what needs to change

*There is a need for programs to support students’ mental and cognitive health*

The consultations revealed a clear need and appetite for new approaches to better support university students’ mental and cognitive health.

Students stated they were struggling with their mental health. Feeling stressed, isolated, burnt out, depressed, anxious and sleep deprived were pervasive and normalised aspects of university life:

*“…we know that pretty much every student is suffering*,* it’s what becomes normalised*,* and what’s become normalised is not sleeping for multiple days to finish off your assignments.” Focus group four*,* female student*.

*“We just need more support. It doesn’t matter what time it is*,* COVID*,* not COVID. Students always need support*,* because we don’t get it enough. Even though we have families and friends*,* uni life is a hard life. It’s hard to manage your life*,* studying*,* maybe working*,* exercising*,* socialising. It’s really hard to manage all of it for anyone*,* especially at this young age.” Focus group two*,* female student*.

International students reported that the absence of familial support and challenges associated with adjusting to a new culture exacerbated their feelings of depression, isolation, and helplessness. These mental health difficulties further diminished their motivation for exercise and impaired their ability to focus:

*“I had depression*,* because I was living alone and I haven’t seen my family in almost two years. As an international student*,* you - you’re feeling all that stuff. You don’t want to exercise. You can’t focus on studies.” Focus group two*,* female student*.

Staff were also concerned about the growing prevalence and impact of mental health issues among students, including depression, anxiety, and stress. They emphasised the academic ramifications of these challenges, noting a marked increase in students’ requests for special consideration and extensions for assignments and exams throughout the year.

Although staff articulated a clear concern for student wellbeing, students were sceptical about the university’s genuine investment in their mental health. This scepticism was fuelled by dissatisfaction with the existing options available to support their mental health at the university, which included once-off wellbeing events and initiatives, such as R U OK? Day, lecture slides that routinely directed students to contact mental health helplines or the medical centre on-campus, and student counselling services. Students described most of these efforts as tokenistic, “box-ticking” gestures designed to fulfill administrative requirements and shift accountability rather than offering genuine sustainable support:

*“…there’s one thing I hate about the units… they start off every week’s content by saying*,* just a reminder*,* if you are having mental health issues*,* please contact these helplines*,* they are there to help blah*,* blah*,* blah… To me*,* it just sounds like*,* okay*,* we just got pressure from our superiors to give you a lot of pressure*,* and if you’re struggling*,* this is someone else’s problem.” Focus group four*,* male student*.

Student counselling services were received positively by a minority of students who found specific counsellors helpful and responsive to their mental health challenges, however, the majority were dissatisfied with the service. Students found it difficult to access an appointment due to long wait times and saw the service as reactive and deficit-based, designed to treat mental health issues after they have already manifested, rather than adopting a proactive, strength-based approach to build resilience against the onset of mental health challenges:

*“…there’s not much like helping I guess like the average student… in terms of counselling services it’s hard to get to… it’s targeted towards like a lot of the stress and in need of help like now. If you’re kind of before that stage*,* it’s really hard to get a counsellor because there’s just so many students and there’s only so many counsellors…getting any help before that point it’s really difficult. I know that I’ve been deterred from getting counselling because of all these wait times…” Focus group three*,* female student*.

International students were particularly disillusioned by the lack of mental health support given their significant financial contributions to the institution. They reported the university treated them like “cash cows”, exploiting their financial investment without providing adequate help in return.

Students reported an absence of programs specifically designed to support their cognitive health; a gap acknowledged by some staff members. Students and staff concurred on the need for meaningful, proactive, and strength-based programs designed to promote sustainable benefits for students mental, cognitive and brain health or, in students’ own words, their “mood, mind and marks”. Students agreed the program messaging and content needed to be positively framed, relevant to their goals, and avoid the stigma surrounding “mental health” terminology:

*“Something to do with mental health sounds very scary. Even if all the population has mental health issues*,* I would still avoid these kind of programs…Even if I’m the one who needs it for example*,* I’ll still be like*,* I’m not a psychopath or I’m not a crazy one…instead of negatively sounding words*,* we should use positively sounding words” Focus group two*,* female student*.

*“…instead of specifically saying mental health*,* it would maybe be better to replace it with mood*,* or a word that is related to mental health that maybe has less stigma. There is a reality where people could read that and subconsciously be turned away from it*,* or consciously*,* either way.” Focus group four*,* female student*.

### Barriers to and facilitators of university students’ exercise

Consultations with students revealed barriers to and facilitators of exercise across five of the six categories of the COM-B model: psychological capability, physical opportunity, social opportunity, automatic motivation, and reflective motivation (see Additional file 5 for example quotes relevant to each theme). No themes emerged in relation to students’ physical capability.

#### Psychological capability

With respect to psychological capability, three themes emerged: knowledge, time management skills, and tracking and receiving feedback.

While students were aware of the broad health benefits of exercise, the majority lacked specific knowledge about the neurophysiological mechanisms that contribute to exercise-induced improvements in mental, cognitive and brain health. Furthermore, they were unaware of the potential for exercise to improve their academic performance, particularly during the exam period. There was consensus among the students that learning this information would motivate them to be more active.

Some students reported they lacked knowledge in exercise domains such as: how to execute proper form and technique while exercising, use exercise equipment, and manage the physical consequences and risks associated with exercise (i.e., muscle soreness, fatigue, and injury). Students noted that it would be helpful to receive instruction and advice from a credible source to address these gaps in their knowledge.

Poor time management skills emerged as a prominent barrier to exercise among students. Transitioning into university was often associated with new academic responsibilities and social opportunities that overwhelmed their ability to allocate time for exercise alongside other commitments. Students described an interest in learning time management skills to help them initiate and maintain a consistent exercise regime.

The majority of students described how tracking and receiving feedback on their exercise progress via digital tools, such as apps and fitness monitors, was motivating, provided the tool was quick and easy to use. However, they held mixed opinions regarding whether it was useful to track and receive feedback on the impact of exercise on their mental health and cognition. Some students believed this type of information would incentivise them to continue to exercise if they gained knowledge of the positive changes in their mental health and cognition, while others were concerned that it would be tedious to track these outcomes in addition to their exercise progress.

#### Physical opportunity

Five themes were identified within physical opportunity: a lack of time, the need for a variety of flexible exercise options, prompts to exercise, financial costs associated with exercise, and the utility of using the university’s digital education platform (i.e., Moodle) to deliver PEAK.

A major barrier to exercise was not having enough time. However, when prompted, some students acknowledged that perceived time constraints were often due to prioritising other activities, such as studying, over exercise, rather than lacking enough time in a day to exercise.

A key facilitator identified by students was the need for choice; they valued the availability of a wide variety of flexible exercise options. Students wanted exercise options that could accommodate their location (i.e., indoors, outdoors, at home, on-campus, and at a gym), variable weather conditions, and preferences regarding exercise delivery (i.e., in-person and digital), type (i.e., aerobic and resistance training), and interpersonal format (i.e., with others and alone). There was a consensus that introductory exercise options aimed at beginners could reduce anxiety associated with being new to a gym or other exercise environment and help them build foundational exercise skills and confidence. Furthermore, there was a preference for exercise modalities that did not require specialised equipment. The collective sentiment was that students wanted PEAK to offer a range of exercise options that could flexibly accommodate any logistical constraints, exercise preference, and skill levels.

The concept of using prompts to facilitate exercise was broadly welcomed. Students believed that timely prompts to exercise could help them to remember to follow through on their intention to exercise, particularly during periods of high stress such as the examination period when they are more likely to forego exercise.

The financial costs associated with engaging in exercise emerged as both a barrier and a facilitator. The cost of gym memberships and exercise programs was generally seen as a barrier to participation. Therefore, most students expressed their preference for PEAK to be free. However, some students felt that making a modest financial commitment could serve as a form of accountability, pushing them to take full advantage of the services for which they have paid.

Students had mixed feelings about using existing university academic content platforms, such as Moodle, for delivering PEAK. Some were sceptical that they would actively engage with the program on Moodle because they often ignore non-academic resources on such a platform. However, other students thought that effective advertising and an engaging program design could counteract this issue and saw advantages in using Moodle given it is frequently accessed for academic content.

#### Social opportunity

Four themes emerged within students’ social opportunities: the impact of exercising with others, receiving encouragement from others to exercise, the impact of exercise role models, and the importance of a supportive exercise community.

Exercising with others yielded multiple advantages for students, enhancing not only their motivation to attend workouts but also their level of physical exertion during these sessions. Committing to exercise with someone also created a sense of accountability. External encouragement to exercise and reminders from friends or partners who were not exercise companions also bolstered students’ commitment to be active. Most students attested that exercising with friends enhanced enjoyment. Exercising with others to meet new people was particularly relevant for students who found it challenging to make friends at university. However, as one student pointed out, preferences for social or solitary exercise could fluctuate within a week, depending on one’s emotional state. Therefore, while the benefits of social exercise are manifold, the availability of choice between group and solitary exercise options was deemed important.

Students indicated a strong desire for exercise role models and champions to promote the adoption of PEAK. Specifically, students expressed a preference for the program to be promoted by people actively involved in student life, rather than university staff who were less relatable and accessible. Hearing transformational stories related to the physical benefits of exercise—especially from a person students could identify with—also served as a powerful motivator. Witnessing or hearing about the tangible benefits that others have gained from regular exercise helped students visualise their own potential for change.

Being part of a supportive and inclusive exercise community had a positive impact on students’ exercise experience. Several students mentioned instances where other more advanced exercisers would proactively offer advice and encouragement to those new to exercise. This support helped to foster a sense of community and inclusivity, making the gym environment less daunting for those new to the gym, and motivating them to return for subsequent sessions.

#### Automatic motivation

Regarding automatic motivation, six themes were identified: feeling tired, poor mental health, feeling self-conscious, enjoyment, having a routine, and receiving incentives.

A common barrier was feeling too tired to exercise. Many students noted that while they may have time to exercise at the end of the day, their energy was significantly depleted, making the prospect of exercise less appealing. The issue of “feeling too tired” was particularly salient during stressful academic periods, where students reported that maintaining a consistent exercise regimen felt almost insurmountable.

Students reported that when their mental health felt poor, such as feeling stressed, sad, or depressed, they were less likely to exercise. Students recognised that the relationship between their mental state and exercise was bidirectional and noted a paradox; while poor mental health often led to a lack of motivation to exercise, the absence of exercise further exacerbated their stress and negative emotional states. This recognition was typically not enough to support action.

Feeling self-conscious, judged, or intimidated while exercising in front of others was a barrier. Many students felt insecure or embarrassed about the way they looked while exercising, particularly if they believed they were being observed by others. For example, some female students indicated feeling uncomfortable or judged while exercising with male friends who made unsolicited comments about their bodies. The fear of making mistakes or appearing inexperienced in the gym setting was another source of intimidation associated with exercise.

Students expressed a strong desire for exercise to be enjoyable. The concept of ‘fun’ was frequently mentioned, with students recalling positive experiences engaging in playful exercises such as dodgeball or listening to music while exercising to enhance enjoyment. Some students found less strenuous exercises, such as walking and cycling, to be more pleasurable than higher-intensity exercise options, whereas other students enjoyed the sense of satisfaction often associated with engaging in physically challenging, higher-intensity exercise options.

Students overwhelmingly reported that the establishment of a consistent routine—encompassing not just exercise but also sleep and diet—played a pivotal role in sustaining their exercise regimen over time. The term “momentum” was recurrently used, indicating that the routine itself served as a self-reinforcing mechanism. The students indicate that the predictability of a set routine reduced the cognitive load associated with planning and decision-making, thereby lowering the psychological barriers to exercise. Students found that once exercise became integrated into their daily routine, it was perceived less as an optional activity and more as a non-negotiable commitment.

The idea of receiving rewards to incentivise their participation in PEAK resonated strongly with students. Various forms of incentives were suggested by students, including: coffee and food vouchers; money; course credit; and, free or discounted access to specialised exercise classes. Students suggested a tiered system of rewards, where meeting specific exercise goals each week or month would lead to a reward.

#### Reflective motivation

Three core themes were identified within reflective motivation: priorities, exercise goals, and beliefs about the impact of exercise.

Students consistently deprioritised exercise in favour of their academic responsibilities. Despite the acknowledgement that exercise could potentially boost students’ academic productivity, many students shared the opinion that the time and energy spent exercising would detract from the time that could be spent studying.

The ability to set and achieve specific exercise goals emerged as a facilitator of sustained exercise. Students emphasised that goals needed to be self-selected, specific, achievable, and personalised to their preferences, exercise skills and fitness level. For those who were new to exercise, starting small and gradually increasing the intensity or duration of exercise made initiating and maintaining a regular exercise routine feel less daunting.

Most students believed that engaging in exercise had a notable positive impact on their mental health and wellbeing. Exercise was considered an effective means of managing stress, offering a psychological ‘buffer’ against the pressures of university life. Some students noted that even shorter sessions of exercise resulted in perceptible mood elevation. Exercise was also seen as a vehicle for positively impacting self-esteem and sense of self-achievement. Notably, the mental health benefits of exercise were observed across a variety of exercise modalities, including walking, running, dancing, team sports and strength and resistance training. However, one student believed that shorter sessions of less intense exercise was more effective in enhancing her mood, as opposed to vigorous, high-intensity workouts, which made her feel unhappy and uncomfortable.

Students reflected on the impact exercise had on their cognition, physical health and appearance. Regarding the cognitive impact, most believed it was easier to concentrate on single tasks and reduce task-switching and mental drift following exercise. However, some found it difficult to think critically and problem-solve directly after engaging in vigorous exercise. The physical impact of exercise also elicited mixed responses. Some students believed exercise increased their energy, while others felt that it expended energy they needed to reserve for academic tasks. Other physical health and aesthetic benefits of exercise, such as improved fitness, weight loss and muscle toning, were additional motivators for maintaining an active lifestyle.

### Barriers to and facilitators of the sustained implementation and scale of PEAK

Staff anticipated barriers to and facilitators of the sustained implementation and scale of PEAK across three of the six categories of the COM-B model: physical opportunity, social opportunity, and reflective motivation (see Additional file 6 for example quotes relevant to each theme). No themes emerged in relation to psychological capability, physical capability, or automatic motivation.

#### Physical opportunity

Staff emphasised the need for PEAK to be affordable for the university and well-resourced. Multiple staff members pointed to financial and operational constraints that could impede the program’s scalability. Any significant extra workload for staff to run the program was noted as a limiting factor. However, several staff identified possible solutions to mitigate these challenges. They suggested that leveraging digital delivery platforms and integrating PEAK with pre-existing university services, such as on-campus exercise facilities and counselling services, could both reduce operational costs and enhance support for the program.

#### Social opportunity

Staff emphasised that the successful adoption and sustainability of PEAK would require a multi-level approach, involving both top-down organisational support and bottom-up student leadership support. Endorsement from senior leadership would lend credibility to the program and was deemed necessary for sustainable resource allocation, program integration, and acceptance within the university. The need for bottom-up support from students to champion PEAK was equally important for gaining students’ trust in the program. Staff felt that messages about PEAK would be better received and accepted if they came from peers, such as student ambassadors, rather than from faculty or administration. Partnerships with local and national external bodies and organisations invested in student wellbeing (e.g., Universities Australia, the National Association of Australian University Colleges and Beyond Blue) could provide an avenue for scaling the program by offering additional resources and a broader platform for program implementation.

#### Reflective motivation

There was consensus that the program would most likely be successful (i.e., adopted and supported) if its goals aligned with the strategic focus of the university. Current university priorities that could be leveraged were supporting students’ mental health (especially in the aftermath of the COVID-19 pandemic), maximising academic performance, and sense of belonging to the university, elevating the university’s reputation for academic excellence, attracting international students to enrol in the university, and reduce institutional financial costs associated with students accessing counselling services or dropping out of university due to poor mental health. Therefore, measuring the impact of the program on these variables was deemed important for garnering long-term support from university leadership and senior staff.

### Data triangulation

Based on the triangulated results of the qualitative research, systematic review, and expert knowledge, thirty-one key barriers and facilitators to university students’ exercise were targeted in the intervention design (Table 3). Barriers and facilitators spanned six COM-B categories: psychological capability (*n* = 7), physical capability (*n* = 1), physical opportunity (*n* = 7), social opportunity (*n* = 4), reflective motivation (*n* = 5) and automatic motivation (*n* = 7).

**Table 3:** Behaviour change wheel process applied to develop the PEAK mood, mind and marks program

*Data source (systematic review/stakeholders/experts)* Student barriers and facilitators to engaging with PEAK	COM-B category	Intervention types	BCT/s	Text description	Mode of delivery	
**“Kick Off session” PEAK program onboarding**						
*Experts.* Students need information about the PEAK program including its purpose, what participation involves, and potential benefits	PSYCH CAP	EDUCATION	Instruction on how to perform a behaviour^a^	Students will be invited to attend a “Kick Off Session” to learn about PEAK’s purpose, how to participate^a^, and how exercise can improve mental and cognitive health^b^.	In-person	
			Information about emotional consequences^b^			
			Information about health consequences^b^			
*Student stakeholders.* Being part of a supportive exercise community is beneficial for students*	SOC OPP	ENABLEMENT	Social support (unspecified)	Students will have the opportunity to meet peer participants and the PEAK program design team at the onboarding “Kick Off Session”.	In-person	
**Menu of exercise options and goal**						
*Systematic review and student stakeholders.* Limited financial means constrain students’ ability to pay for exercise options* Students need [a range of] accessible exercise options that can flexibly accommodate their location, weather conditions, and exercise preferences* Students want exercise options that can flexibly cater to their preference for exercising alone or with others*	PHYS OPP	ENABLEMENT	Restructuring the physical environment	Each week, students will be given access to a new menu of free exercise options that comprise: aerobic and resistance training options; beginner and advanced options; digital and in-person training options; options that do not require exercise equipment.	Digitally via Moodle	
*Student stakeholders.* Students hold mixed opinions on whether it is helpful/unhelpful to use existing university digital platforms like Moodle to deliver the program Beginners need introductory exercise options to build confidence and learn basic exercise skills Students need exercise options that don’t require the use of equipment						
*Systematic review and student stakeholders.* Students need [a range of] accessible exercise options that can flexibly accommodate their location, weather conditions, and exercise preferences* Limited financial means constrain students’ ability to pay for exercise options* Students want exercise options that can flexibly cater to their preference for exercising alone or with others*	PHYS OPP	ENABLEMENT	Adding objects to the environment	Students will be given an "exercise starter kit" which includes an exercise mat, drink bottle and towel to enable online exercise options at home, a calendar to schedule their exercise sessions, and 12 free passes to access the university gym and/or group exercise sessions on-campus.	In-person	
*Systematic review and experts.* Receiving advice from trusted experts or credible sources regarding exercise is motivating.	REF MOT	PERSUASION	Credible source	Students will be informed that the exercise options were curated by a senior exercise physiologist.	Digitally via Moodle	
*Systematic review and student stakeholders.* Students lack knowledge about how to exercise	PSYCH CAP	EDUCATION	Instruction on how to perform a behaviour^a^	Fitness and/or gym instructors will provide instruction^a^ and demonstration^b^ of how to perform various exercises while prompting students to engage in same^c^.	Digitally via Moodle and in-person	
*Systematic review.* Having the physical skills to engage in exercise is important for students	PHYS CAP	TRAINING	Demonstration of the behaviour^b^			
			Behavioural practice/rehearsal^c^			
*Systematic review and student stakeholders.* Students find it motivating to set exercise goals	REF MOT	ENABLEMENT	Goal setting (behaviour)^a^	Students will be informed that the core PEAK program exercise goal is to build up to exercising three or more times per week for 12-weeks^a^.	Digitally via Moodle and in-person	
			Action planning^a^			
**Twelve weekly educational and motivational videos**						
*Systematic review and student stakeholders.* Having a routine helps students to fit exercise into their schedule*	AUTO MOT	ENVIRONMENTAL RESTRUCTURING	Prompts/cues^a^	***Week 1. “Expert tips for creating an exercise routine”*** The following expert-endorsed strategies for creating an exercise routine will be depicted: 1) Visualise desired exercise outcomes and place reminders of this in visible locations^a^ 2) Use a calendar to plan exercise sessions in advance^b^ 3) Track weekly exercise sessions^c^ 4) Create "IF this, THEN that" exercise plans to overcome foreseeable barriers to exercise^a^ 5) Start with small exercise activities that can piggyback off existing routines and habits^a^	Digitally via Moodle	
		ENABLEMENT	Goal setting (behaviour)^b^			
			Action planning^b^			
			Self-monitoring of behaviour^c^			
*Systematic review and student stakeholders.* Students lack time management skills to fit exercise into their routine	PSYCH CAP	EDUCATION	Not specified			
*Student stakeholders.* Students are motivated by the positive impact that exercise has on their thinking skills*	REF MOT	EDUCATION	Information about health consequences	***Week 2. “Too many assignments to exercise? Exercising your way to PEAK marks”*** A student struggling to concentrate, remember things, and problem-solve will be shown. Evidence regarding how exercise can improve these three cognitive functions will then be provided.	Digitally via Moodle	
		PERSUASION				
*Systematic review and student stakeholders.* Students may feel self-conscious when exercising around others	AUTO MOT	PERSUASION	Framing/reframing	***Week 3. “Overcoming self-consciousness”*** Feeling self-conscious while exercising will be normalised and students will be encouraged to adopt a new and empowering perspective to embrace the way their body looks and feels while exercising rather than feeling self-conscious. These messages will be shown alongside rolling footage of culturally, gender, and age diverse students exercising together.	Digitally via Moodle	
*Systematic review and student stakeholders.* Students can find it difficult to prioritise exercise over other activities*	REF MOT	PERSUASION	Framing/reframing	***Week 4. “But what if I don’t have time to exercise?”*** A psychologist will reframe the belief that "I don’t have enough time to exercise" to "I don’t prioritise exercise", and encourage students to re-prioritise exercise by remembering why they signed up to the PEAK program.	Digitally via Moodle	
*Systematic review and student stakeholders.* Students are motivated by the positive impact that exercise has on their mental health and wellbeing*	REF MOT	EDUCATION	Information about emotional consequences^a^	***Week 5. “Too stressed to exercise? Exercising your way to PEAK mood”*** A stressed and inactive student with low mood and low energy will be shown in the context of relatable student life. This will be followed by footage of the same student using exercise to cope with stress, improve their mood^a^ and increase their energy^b^. Education regarding the mental health benefits of exercise will be introduced alongside this footage^a^.	Digitally via Moodle	
			Information about health consequences^b^			
*Systematic review and student stakeholders.* Students don’t feel like exercising when they are tired* Students don’t feel like exercising when their mental health is poor*	AUTO MOT	PERSUASION	Information about emotional consequences^a^			
			Information about health consequences^b^			
*Student stakeholders.* Knowledge of how exercise impacts mental, cognitive and brain health is helpful*	PSYCH CAP	EDUCATION	Information about emotional consequences^a^	***Week 6. “Strong lungs, strong brain: the beauty of breathlessness”*** An animation will be used to explain the neurophysiological processes underlying how training at higher intensities can improve mental^a^ and brain health^b^. An example of how breathlessness can be used as a practical way of assessing exercise intensity will also be provided^b^.	Digitally via Moodle	
			Information about health consequences^b^			
*Student stakeholders.* Knowledge of how exercise impacts mental, cognitive and brain health is helpful*	PSYCH CAP	EDUCATION	Information about health consequences	***Week 7. “Strong body, strong brain: the beauty of strength training”*** An animation will be used to explain the neurophysiological processes underlying how strength and resistance training can improve brain health and cognitive health.	Digitally via Moodle	
*Systematic review and student stakeholders.* Students can find it difficult to prioritise exercise over other activities*	REF MOT	PERSUASION	Valued self-identity	***Week 8. “Connecting with your WHY”*** A psychologist will facilitate a self-reflection exercise for students, designed to help them identify the specific benefits they seek from physical activity and to explore how these benefits align with their core values.	Digitally via Moodle	
*Systematic review.* Students dislike experiencing discomfort during exercise	AUTO MOT	PERSUASION	Information about health consequences^a^	***Week 9. “Expert tips for sustaining an exercise routine”*** The following expert-endorsed strategies for sustaining an exercise routine will be depicted: 1) Get comfortable with being uncomfortable; pushing beyond your comfort zone results in the biggest benefits of exercise^a^; remind yourself the effort is short but the rewards are long-lasting^b^ 2) Create accountability by exercising with others^c^ or booking an exercise class in advance^d^ 3) Expect to miss sessions sometimes, but don’t let lapses stop you	Digitally via Moodle	
			Self-talk^b^			
*Systematic review and student stakeholders.* Having a routine helps students to fit exercise into their schedule*		ENABLEMENT	Social support (unspecified)^c^ Action planning^d^			
*Systematic review and student stakeholders.* Having a routine helps students to fit exercise into their schedule*	AUTO MOT	PERSUASION	Framing/reframing^a^	***Week 10. “Consistency trumps perfection; why habits are the gold”*** Experiencing setbacks in maintaining an exercise routine will be normalised, and students will be encouraged to adopt the perspective that “doing something is better than nothing”^a^ with regards to long-term maintenance of consistent exercise habits^b^.	Digitally via Moodle	
			Information about health consequences^b^			
*Systematic review and student stakeholders.* Students are motivated by the positive impact that exercise has on their mental health and wellbeing*	REF MOT	EDUCATION	Information about emotional consequences^a^	***Week 11. “But what if I don’t feel like exercising?!”*** A student staying at home on the couch, feeling “too tired, stressed and flat” to exercise, will be shown alongside the negative consequence that skipping exercise has on reinforcing unhealthy habits^b^. The same student will overcome their fatigue by engaging in exercise, subsequently improving their mood^a^, increasing their satisfaction and maintaining their exercise habits^b^.	Digitally via Moodle	
			Information about health consequences^b^			
*Systematic review and student stakeholders.* Students don’t feel like exercising when they are tired* Students don’t feel like exercising when their mental health is poor*	AUTO MOT	PERSUASION	Information about emotional consequences^a^			
			Information about health consequences^b^			
*Student stakeholders.* Students are motivated by the positive impact that exercise has on their thinking skills*	REF MOT	PERSUASION	Framing/reframing	***Week 12. “SWOTVAC is coming... keep moving!”*** A student debating whether to exercise during their exam preparation week (SWOTVAC) will be shown. During this debate, information will be provided regarding how exercise can help students better manage their stress, improve their thinking skills and ultimately help (rather than hinder) their exam performance.	Digitally via Moodle	
*Systematic review and student stakeholders.* Students can find it difficult to prioritise exercise over other activities*						
**"PEAK Points" reward system**						
*Systematic review and student stakeholders.* Students respond positively to incentives that encourage them to exercise	AUTO MOT	INCENTIVISATION	Material incentive (behaviour)^a^	Students will receive notification that they are eligible to earn one ‘PEAK Point’ for each completed exercise session^a^. These accrued points may be redeemed for either on-campus coffee vouchers^b^ or for personalized small group training sessions led by a senior exercise physiologist^c^	Digitally via text message	
			Non-specific incentive (behaviour)^a^			
			Material reward (behaviour)^b^			
			Non-specific reward (behaviour)^c^			
**WhatsApp group**						
*Systematic review and student stakeholders.* Encouragement from others to exercise helps students to keep accountable to their exercise routine Students respond positively to relatable exercise role models and champions who support and encourage exercise*	SOC OPP	ENABLEMENT	Social support (unspecified)	Students will receive encouragement to exercise from the PEAK design team, including two PEAK student ambassadors.	Digitally via WhatsApp	
*Student stakeholders.* Being part of a supportive exercise community is beneficial for students*						
*Systematic review and student stakeholders.* Students respond positively to relatable exercise role models and champions who support and encourage exercise*	SOC OPP	MODELLING	Credible source	Two PEAK student ambassadors will share their exercise journey and experiences with students.	Digitally via WhatsApp	
*Student stakeholders.* Students lack knowledge about how to manage the physical consequences (e.g., muscle soreness) and risks (e.g., injury) associated with exercise	PSYCH CAP	EDUCATION	Information about health consequences	A senior exercise physiologist will share two educational videos of himself titled ***“The truth about delayed onset muscle soreness (DOMS)”*** and ***“Managing exercise fatigue and recovery”.*** During these videos, he will explain how to manage the physical consequences of exercise (e.g., muscle soreness and fatigue), prevent injury, and optimise recovery following exercise.	Digitally via WhatsApp	
*Systematic review.* Students dislike the unpleasant physical consequences of exercise, such as muscle soreness and fatigue	AUTO MOT					
*Systematic review and experts.* Students’ beliefs in their ability to exercise and perceiving it as a part of their identity is important	REF MOT	PERSUASION	Credible source^a^	A senior exercise physiologist^a^ will share a motivational video of himself titled ***“Busting exercise myths”***. During this video, he will target students’ self-efficacy to exercise by challenging misconceived beliefs that people need to be “good” at exercise and/or self-identify as “sporty” to reap the benefits of exercise^b^.	Digitally via WhatsApp	
			Verbal persuasion about capability^b^			
**"PEAK Packs" exercise groups**						
*Systematic review and student stakeholders.* Exercising with others is beneficial for students	SOC OPP	ENVIRONMENTAL RESTRUCTURING	Restructuring the social environment	Students will have the option to exercise with others in the PEAK program either with a pre-selected group of friends or by joining a curated group of fellow participants, termed a “PEAK Pack”. Within these PEAK Packs, students will be integrated into dedicated WhatsApp sub-groups, which operate alongside the main WhatsApp group encompassing all participants.	In-person and digitally via WhatsApp	
			Social support (unspecified)	Students will be encouraged to exercise with others in their PEAK Pack and to support each other’s exercise engagement.		
**Exercise and wellbeing trackers**						
*Student stakeholders.* Prompts to exercise are a helpful reminder for students	PHYS OPP	ENVIRONMENTAL RESTRUCTURING	Prompts/cues^a^	Students will be sent an “Exercise Tracker” each week^a^ to track the number, type and duration of exercise they complete^b^.	Digitally via Qualtrics link embedded in text message	
*Systematic review and student stakeholders.* Tracking and receiving feedback on exercise progress is motivating for students*	PSYCH CAP	EDUCATION	Self-monitoring of behaviour^b^			
*Student stakeholders.* Students hold mixed opinions on whether it is/is not helpful to track and receive feedback on the impact of exercise on mental health and cognition*	PSYCH CAP	EDUCATION	Self-monitoring of outcome(s) of behaviour^a^	Students will be sent a “Wellbeing Tracker” tri-weekly to track the impact exercise has^a^ on their wellbeing and cognition^b^.	Digitally via Qualtrics link embedded in text message	
			Monitoring of emotional consequences^b^			
**Program outcome reports**						
*Systematic review and student stakeholders.* Tracking and receiving feedback on exercise progress is motivating for students*	PSYCH CAP	EDUCATION	Feedback on behaviour^a^	A personalised “PEAK Outcome Report” will be provided to each student at the conclusion of PEAK. This report will comprise: the number of days they exercised per week^a^; the total number of minutes spent exercising^a^; and their mental, and cognitive health outcomes^b^.	Digitally via email	
		PERSUASION				
*Student stakeholders.* Students hold mixed opinions on whether it is/is not helpful to track and receive feedback on the impact of exercise on mental health and cognition*	PSYCH CAP	EDUCATION	Feedback on outcome(s) of behaviour^b^			
		PERSUASION				

*Note.* *Barrier/facilitator to exercise targeted in multiple aspects of the program. Psych Cap: Psychological Capability; Phys Cap: Physical Capability; Phys Opp: Physical Opportunity; Soc Opp: Social Opportunity; Ref Mot: Reflective Motivation; Auto Mot: Automatic Motivation

### Stage 2: identify intervention options

#### Step 5 and 6: identify intervention types and policy options

Seven intervention types (Table 3) were selected for the program based on the APEASE criteria: education, modelling, persuasion, environmental restructuring, incentivisation, training and enablement (see Additional file 1 for definitions of intervention types). The intervention types ‘coercion’ and ‘restriction’ were excluded because they did not satisfy the APEASE criteria. Specifically, coercion was not deemed acceptable or appropriate by university students and staff (acceptability) and bore the risk of unintended consequences, such as student disengagement from the program and adverse impacts on their wellbeing (side-effects/safety). Restriction did not satisfy the criteria because it was not practical to reduce the opportunity for students to engage in competing behaviours (practicability).

### Stage 3: identify intervention content and delivery mode

#### Step 7 and 8: identify behaviour change techniques and mode of delivery

Twenty-six BCTs were selected to enact the relevant intervention types (Table 3, see Additional file 7 for definitions of each BCT). The content and mode of delivery for each BCT was operationalised into eight core program components:

1. A “Kick Off session” for program onboarding,
2. A new menu of free exercise options each week curated by an exercise physiologist (SH) and exercise goal,
3. Twelve weekly educational and motivational videos (view videos online at https://youtube.com/playlist?list=PLHKkW53lhmzRRKdL8M5TyBoZiIAm-WrwY&si=akmm0QjyXtQbV5So),
4. “PEAK Points” reward system,
5. WhatsApp social support group,
6. “PEAK Packs” exercise group,
7. Exercise and wellbeing trackers,
8. Program outcome reports.

These eight core components were designed to be delivered over multiple digital channels and a small number of in-person activities. Digital channels included Moodle, WhatsApp, Qualtrics, text message, and email. These channels were specifically chosen for their cost-effectiveness, to adhere to budget constraints during the development phase and to maximise the program’s potential for scale.

The program duration was set at 12-weeks to coincide with the full academic semester at Monash University. The sequence of educational and motivational video content was strategically ordered by the design team to correspond with the typical fluctuation in students’ motivation to exercise throughout the semester.

## Discussion

This research is the first to describe how the BCW was applied to develop PEAK, a multichannel intervention designed to engage university students in three or more exercise sessions per week for the benefit of their mental and cognitive health. The triangulated findings from the stakeholder consultations, systematic review, and expert consensus identified 31 barriers and facilitators affecting students’ capability, opportunity, and motivation to engage in exercise. Seven intervention types were identified as relevant and subsequently mapped onto 26 BCTs.

Students confirmed a clear need for mental health support and highlighted an absence of programs to adequately address these concerns. These findings call for sustainable, proactive programs within the university setting that can concurrently support students’ mental and cognitive health. Harnessing the well-established mental and cognitive health benefits of exercise [8, 9, 40] offers a solution to address this unmet need. This approach has demonstrated value in numerous other contexts and populations. For example, integrating exercise as a routine part of psychiatric care has gained considerable attention in recent years due to the growing body of evidence supporting its efficacy in treating a range of mental disorders [41]. Similarly, workplace wellness interventions are increasingly focused on improving the mental wellbeing and productivity of employees by implementing strategies to increase physical activity and reduce sedentary behaviour [42, 43]. These examples demonstrate the potential of adopting exercise-based approaches, such as PEAK, in the tertiary education sector to offer similar mental and cognitive health benefits for students. Targeting university students is particularly important, as it provides an opportunity to set young adults up for a lifetime of healthy exercise habits, establishing a foundation for mentally healthy and productive communities and workforces.

University staff expressed a strong interest in endorsing programs that align with institutional goals, such as supporting student wellbeing and academic performance, advancing the university’s reputation for academic excellence, increasing student enrolment, and strengthening students’ sense of belonging to the university. A program that can successfully support students’ mental and cognitive health aligns well with these institutional goals. Such a program has the potential to enhance the student experience and improve their academic performance, thereby promoting the university’s academic reputation on a competitive global stage. A strong reputation can lead to higher world university rankings and increased visibility in the academic community, which can attract academically talented students, domestically and internationally, to enrol in the university. This holds significance given the recent decline in international student enrolment in universities across several countries [44–46], which has consequential adverse economic ramifications [47]. Therefore, strategies to reverse this trend are increasingly important for universities.

Of equal importance is a program that can strengthen students’ sense of belonging to the university. A strong sense of belonging can increase students’ academic motivation, enjoyment of their studies, and engagement with the university [48, 49], all of which heavily influence institutional performance indicators such as academic success and student retention rates [50–52]. The need for interventions that address students’ sense of belonging is particularly relevant following the COVID-19 pandemic, which restricted students’ opportunities for peer interaction and engagement with the university community. Interventions that aim to foster students’ sense of belonging to the university should focus on enhancing peer-to-peer interaction [53]. PEAK was designed with this specific goal in mind, offering students opportunities to connect with each other and build a community of like-minded peers via their participation in exercise and shared experience of the program.

### Strengths and limitations

This study provides a thorough example of how to apply the BCW to integrate theory, evidence, stakeholder perspectives, and expert knowledge into the development of a health behaviour change intervention. Describing this process in detail enables other intervention designers to pilot the intervention with fidelity to test its possible impact across various other university settings. The methodology aligns with the MRC guidelines and the NICE recommendations, thereby ensuring adherence to best practice principles for intervention development. Another strength lies in the purposeful recruitment of students and staff in the stakeholder engagement process. This recruitment strategy enabled a diversity of perspectives to be captured and addressed in the intervention design. However, there are limitations to consider. Using the BCW process can be time-intensive, which may pose challenges for the rapid development and implementation of interventions. Moreover, the absence of students with physical mobility limitations in the sample could partially account for the lack of identified barriers or facilitators related to physical capability in the qualitative data. Lastly, a bout of exercise was defined as 10 min or more of exercise. This definition was chosen to enable students to track and log their participation in exercise and encourage them to engage in intentional exercise beyond incidental physical activity associated with daily activities. However, given shorter bouts of physical activity is also associated with health benefits [54], future iterations and evaluations of PEAK could incorporate passive sensing technology to capture all levels of physical activity.

## Conclusion

Behaviour change interventions have the potential to embed regular exercise into the lifestyles of university students to improve their mental and cognitive health. The current study describes how PEAK was developed, and the evidence and stakeholder perspectives used to inform its creation. Using this systematic approach was demonstrated to be effective at translating BCTs into the core components of PEAK, and optimising its feasibility, adoption, and scalability potential. This work offers a replicable roadmap for others seeking to develop behaviour change interventions and will inform an empirical study assessing the efficacy and implementation of PEAK.

### Electronic supplementary material

Below is the link to the electronic supplementary material.

Supplementary Material 1

Supplementary Material 2

Supplementary Material 3

Supplementary Material 4

Supplementary Material 5

Supplementary Material 6

Supplementary Material 7

## Data Availability

The datasets used and/or analysed during the current study and materials used are available from the corresponding author on reasonable request.

## Abbreviations

APEASE: Affordability, Practicability, Effectiveness/cost-effectiveness, Acceptability, Side-effects/safety, Equity
BCW: Behaviour Change Wheel
BCT: Behaviour Change Technique
BCTTv1: Behaviour Change Technique Taxonomy Version 1
COM-B: Capability, Opportunity, Model-Behaviour
MRC: Medical Research Council
NICE: National Institute for Health and Care Excellence
PEAK: PEAK Mood, Mind, and Marks program

## Footnotes

1 The program title developed through consultations with students to create an engaging and appealing name that reflected their understanding of ‘mental health, cognitive health, and brain health’. The longer title was abbreviated to “PEAK” to simplify communication and emphasise the program’s goal of helping students to reach their “peak” performance.
