# Supplementary material for: Developing the PEAK mood, mind, and marks program to support university students’ mental and cognitive health through physical exercise: a qualitative study using the Behaviour Change Wheel
Source: BMC Public Health. 2024 Jul 23;24:1959. doi: 10.1186/s12889-024-19385-x (PMC11265317; doi:10.1186/s12889-024-19385-x)
Supplement: Supplementary file 1 — Supplementary Material 1 [file 12889_2024_19385_MOESM1_ESM.docx]

## **Additional File 6.**

## University Staff and Senior Leadership Anticipated Barriers and Facilitators to the Adoption and Scalability of PEAK

| **COM-B category** | **Theme/ belief statement** | **Sub-theme/belief statement** | **Barrier/facilitator/mixed** | **Example quote(s)** |
| --- | --- | --- | --- | --- |
| CAPABILITY | | | | |
| Psy C | None identified | |  |  |
| Phys C | None identified | |  |  |
| OPPORTUNITY | | | | |
| Phys O | PEAK needs to be affordable for the university and well-resourced | | Barrier | *“What would hinder it’s [scalability]? Well, there's cost of service delivery for us, so there would need to be some form of - and I'm just thinking out loud here - there would probably need to be some form of recompense, or funding attached to it.”* University exercise facilities  *“Having [PEAK] well-resourced I think would be an important thing.”* (Residential services and colleges)  *“with the extra workload for staff to deliver the program if that’s how it would be integrated I think that would probably be the biggest problem there is”* Unit co-ordinator |
|  | Integrating the design and delivery of PEAK with pre-existing university services and platforms will reduce operational costs and enhance the universities long-term support of PEAK | | Facilitator | *“In terms of the design of it, if you were starting with a blank piece of paper, you'd want to have the integration of the services that are available to the students included. So you'd want to have counselling, you'd want to have the physical activity programs we deliver, you'd want to have in person and digital”* University exercise facilities |
| Soc O | Support from others to champion PEAK is important | Local bottom-up student leadership support | Facilitator | *“When it comes from us it’s just – it’s not authentic enough. We can tell students all day that this is important and you should do this to look after yourself but because we’re not in their shoes, we’re not in the same position as them, I don’t think it is as well received as if it comes from people that are either in their peer group or just one or two steps above them.”* Unit co-ordinator |
|  |  | Organisational top-down leadership support | Facilitator | *“You need all the stakeholders buy-in…a top-down approach is really important.”* International student engagement |
|  | Working with partner organisations will enhance the scalability of PEAK | | Facilitator | *“…get a larger organisation like NAAUC [National Association of Australian University Colleges] to give it credibility and what not, and then through discussion about what it is et cetera. Then that will just percolate through to the colleges I’d imagine.”* Residential services and colleges |
| MOTIVATION | | | | |
| Auto M | None identified |  |  |  |
| Ref M | The goals of PEAK need to align with organisational priorities, goals, and policies | The university values programs that enhance student wellbeing and academic performance | Facilitator | *“If [PEAK] were framed as a strengths based or skill building program that was linked somehow to their academic performance that might be a good way to get some buy in.”* (Unit co-ordinator)  *“Student engagement [is an important program outcome]. How many students get involved, reductions in isolation or alienation. I guess, just hearing about happy students and what they got out of the program.”* International student engagement |
|  |  | The university values programs that enhance students’ sense of belonging to university | Facilitator | *“it has started to become really important for the university, in terms of a sense of belonging, for students. Not coming to campus and seeing Monash as just where I go to get to learn the curriculum for my course.”* University exercise facilities |
|  |  | The university values programs that attract talented students to the university | Facilitator | *“I think the ultimate KPI, the ultimate measure is, do talented students want to come to Monash? That's the ultimate. Have we got a university which genuinely cares enough about the students for us to be a university of choice?”* University exercise facilities |
|  |  | The university values programs that reduce institutional financial costs | Facilitator | *“Well, the university probably looks at anything that’s going to reduce costs, so clearly, if this reduces the number of students going to counselling for example…I think that could be a selling point”* Residential services and colleges |

*Note.* Psy C, psychological capability; Phys C, physical capability; Phys O, physical opportunity; Soc O, social opportunity; Ref M, reflective motivation; Auto M, automatic motivation
