# Supplementary material for: Developing the PEAK mood, mind, and marks program to support university students’ mental and cognitive health through physical exercise: a qualitative study using the Behaviour Change Wheel
Source: BMC Public Health. 2024 Jul 23;24:1959. doi: 10.1186/s12889-024-19385-x (PMC11265317; doi:10.1186/s12889-024-19385-x)
Supplement: Supplementary file 2 — Supplementary Material 2 [file 12889_2024_19385_MOESM2_ESM.docx]

**Additional File 3.**

Staff Semi-structured Interview Schedule

**Introduction**

- Introduce PEAK, work to date, ultimate aims,
- Describe purpose of interviews generally, and for interviewing them specifically,
- Obtain informed consent.

**Document interviewee and department’s role**

1.  Can you briefly tell me about your role at Monash?

a.   What does it involve? How long have you been in the role?

2. Can you tell me what the key role and goals of your department / service are within the university?

**Departments perspective and role (if any) in student mental health and cognitive health**

3. Is student mental health and cognitive health a focus in your department?

1. Are there strategies in place to support students’ mental and cognitive health? Can you describe them?
2. What has helped or got in the way of their success?

*(probe department initiation and support + student adoption)*

4. How do you think students are doing from mental health and cognitive health perspectives?

**Department’s perspective on PEAK’s worth and scalability**

5. Do you think PEAK would be beneficial for i) students and your ii) department?

6. Do you think PEAK could be implemented at Monash, supported by the staff and taken up by the students?

a. What do you think needs to be in place for that to happen?

- prompt Capability (knowledge / skills)

- prompt Opportunity (physical / social)

- prompt Motivation (reflective / automatic)

7. If the pilot is successful, what chance do you think PEAK has of being scaled up across Monash and to other universities?

1. What do you think would help or get in the way of its scalability?

**Department’s potential involvement in PEAK**

8. Would you, and your department, want to be involved?

9. What things would you need to know, and what would you need to have in place, to make your department's contribution successful?

1. What processes or other factors would get in the way of your involvement?
2. What processes or other factors would help your involvement?

10. In your department, who do we need to win over, in addition to your good self?

**Co-design**

11. What would you like to see included in the PEAK program?

12. What outcomes would you like to see measured from PEAK, what outcomes would be useful for your department?

1. Do you think measuring these things would help scaling the program up across Monash and to other universities? Why?

**Conclusion**

And finally, are there any other comments you would like to make about PEAK, your department, or questions I have asked today?
