# Supplementary material for: Developing the PEAK mood, mind, and marks program to support university students’ mental and cognitive health through physical exercise: a qualitative study using the Behaviour Change Wheel
Source: BMC Public Health. 2024 Jul 23;24:1959. doi: 10.1186/s12889-024-19385-x (PMC11265317; doi:10.1186/s12889-024-19385-x)
Supplement: Supplementary file 3 — Supplementary Material 3 [file 12889_2024_19385_MOESM3_ESM.docx]

**Additional File 7.**

Behaviour Change Techniques Selected for PEAK, Definitions, and Examples

| **No.** | **Behaviour Change Technique** | **Definition** | **Examples** |
| --- | --- | --- | --- |
| 1.4 | Action planning | Prompt detailed planning of performance of the behaviour (must include at least one of context, frequency, duration and intensity). Context may be environmental  (physical or social) or internal (physical,  emotional or cognitive) (includes ‘Implementation Intentions’)  Note: evidence of action planning does not necessarily imply goal setting, only code latter if sufficient evidence | Encourage a plan to carry condoms when going out socially at weekends  Prompt planning the performance of a particular physical activity (e.g. running) at a particular time (e.g. before work) on certain days of the week |
| 12.5 | Adding objects to the environment | Add objects to the environment in order to facilitate performance of the behaviour Note: Provision of information (e.g. written, verbal, visual) in a booklet or leaflet is insufficient. If this is accompanied by social support, also code 3.2, Social support (practical); if the environment is changed beyond the addition of objects, also code 12.1, Restructuring the physical environment | Provide free condoms to facilitate safe sex  Provide attractive toothbrush to improve tooth brushing technique |
| 8.1 | Behavioural practice/rehearsal | Prompt practice or rehearsal of the performance of the behaviour one or more times in a context or at a time when the performance may not be necessary, in order to increase habit and skill Note: if aiming to associate performance with the context, also code 8.3, Habit formation | Prompt asthma patients to practice measuring their peak flow in the nurse’s consulting room |
| 9.1 | Credible source | Present verbal or visual communication from a credible source in favour of or against the behaviour Note: code this BCT if source generally agreed on as credible e.g., health professionals, celebrities or words used to indicate expertise or leader in field and if the communication has the aim of persuading; if information about health consequences, also code 5.1, Information about health consequences, if about emotional consequences, also code 5.6, Information about emotional consequences; if about social, environmental or unspecified consequences also code 5.3, Information about social and environmental consequences | Present a speech given by a high status professional to emphasise the importance of not exposing patients to unnecessary radiation by ordering x-rays for back pain |
| 6.1 | Demonstration of the behaviour | Provide an observable sample of the performance of the behaviour, directly in person or indirectly e.g. via film, pictures, for the person to aspire to or imitate (includes ‘Modelling’). Note: if advised to practice, also code, 8.1, Behavioural practice and rehearsal; If provided with instructions on how to perform, also code 4.1, Instruction on how to perform the behaviour | Demonstrate to nurses how to raise the issue of excessive drinking with patients via a role-play exercise |
| 2.2 | Feedback on behaviour | Monitor and provide informative or evaluative feedback on performance of the behaviour (e.g. form, frequency, duration, intensity) Note: if Biofeedback, code only 2.6, Biofeedback and not 2.2, Feedback on behaviour; if feedback is on outcome(s) of behaviour, code 2.7, Feedback on outcome(s) of behaviour; if there is no clear evidence that feedback was given, code 2.1, Monitoring of behaviour by others without feedback; if feedback on behaviour is evaluative e.g. praise, also code 10.4, Social reward | Inform the person of how many steps they walked each day (as recorded on a pedometer) or how many calories they ate each day (based on a food consumption questionnaire) |
| 2.7 | Feedback on outcome(s) of behaviour | Monitor and provide feedback on the outcome of performance of the behaviour Note: if Biofeedback, code only 2.6, Biofeedback and not 2.7, Feedback on outcome(s) of behaviour; if feedback is on behaviour code 2.2, Feedback on behaviour; if there is no clear evidence that feedback was given code 2.5, Monitoring outcome(s) of behaviour by others without feedback; if feedback on behaviour is evaluative e.g. praise, also code 10.4, Social reward | Inform the person of how much weight they have lost following the implementation of a new exercise regime |
| 13.2 | Framing/reframing | Suggest the deliberate adoption of a perspective or new perspective on behaviour (e.g. its purpose) in order to change cognitions or emotions about performing the behaviour (includes ‘Cognitive structuring’); If information about consequences then code 5.1, Information about health consequences, 5.6, Information about emotional consequences or 5.3, Information about social and environmental consequences instead of 13.2, Framing/reframing | Suggest that the person might think of the tasks as reducing sedentary behaviour (rather than increasing activity) |
| 1.1 | Goal setting (behaviour) | Set or agree on a goal defined in terms of the behaviour to be achieved Note: only code goal-setting if there is sufficient evidence that goal set as part of intervention; if goal unspecified or a behavioral outcome, code 1.3, Goal setting (outcome); if the goal defines a specific context, frequency, duration or intensity for the behaviour, also code 1.4, Action planning | Agree on a daily walking goal (e.g. 3 miles) with the person and reach agreement about the goal  Set the goal of eating 5 pieces of fruit per day as specified in public health guidelines |
| 5.6 | Information about emotional consequences | Provide information (e.g. written, verbal, visual) about emotional consequences of performing the behaviour Note: consequences can be related to emotional health disorders (e.g. depression, anxiety) and/or states of mind (e.g. low mood, stress); not including 5.5, Anticipated regret; consequences can be for any target, not just the recipient(s) of the intervention; if information about health consequences code 5.1, Information about health consequences; if about social, environmental or unspecified code 5.3, Information about social and environmental consequences | Explain that quitting smoking increases happiness and life satisfaction |
| 5.1 | Information about health consequences | Provide information (e.g. written, verbal, visual) about health consequences of performing the behaviour Note: consequences can be for any target, not just the recipient(s) of the intervention; emphasising importance of consequences is not sufficient; if information about emotional consequences, code 5.6, Information about emotional consequences; if about social, environmental or unspecified consequences code 5.3, Information about social and environmental consequences | Explain that not finishing a course of antibiotics can increase susceptibility to future infection  Present the likelihood of contracting a sexually transmitted infection following unprotected sexual behaviour |
| 4.1 | Instruction on how to perform a behaviour | Advise or agree on how to perform the behaviour (includes ‘Skills training’) Note: when the person attends classes such as exercise or cookery, code 4.1, Instruction on how to perform the behaviour, 8.1, Behavioral practice/rehearsal and 6.1, Demonstration of the behaviour | Advise the person how to put a condom on a model of a penis correctly |
| 10.1 | Material incentive (behaviour) | Inform that money, vouchers or other valued objects will be delivered if and only if there has been effort and/or progress in performing the behaviour (includes ‘Positive reinforcement’) Note: if incentive is social, code 10.5, Social incentive if unspecified code 10.6, Non-specific incentive, and not 10.1, Material incentive (behaviour); if incentive is for outcome, code 10.8, Incentive (outcome). If reward is delivered also code one of: 10.2, Material reward (behaviour); 10.3, Non-specific reward; 10.4, Social reward, 10.9, Self-reward; 10.10, Reward (outcome) | Inform that a financial payment will be made each month in pregnancy that the woman has not smoked |
| 10.2 | Material reward (behaviour) | Arrange for the delivery of money, vouchers or other valued objects if and only if there has been effort and/or progress in performing the behaviour (includes ‘Positive reinforcement’) Note: If reward is social, code 10.4, Social reward, if unspecified code 10.3, Nonspecific reward, and not 10.1, Material reward (behaviour); if reward is for outcome, code 10.10, Reward (outcome). If informed of reward in advance of rewarded behaviour, also code one of: 10.1, Material incentive (behaviour); 10.5, Social incentive; 10.6, Non-specific incentive; 10.7, Self-incentive; 10.8, Incentive (outcome) | Arrange for the person to receive money that would have been spent on cigarettes if and only if the smoker has not smoked for one month |
| 5.4 | Monitoring of emotional consequences | Prompt assessment of feelings after attempts at performing the behaviour | Agree that the person will record how they feel after taking their daily walk |
| 10.6 | Non-specific incentive (behaviour) | Inform that a reward will be delivered if and only if there has been effort and/or progress in performing the behaviour (includes ‘Positive reinforcement’) Note: if incentive is material, code 10.1, Material incentive (behaviour), if social, code 10.5, Social incentive and not 10.6, Non-specific incentive; if incentive is for outcome code 10.8, Incentive (outcome). If reward is delivered also code one of: 10.2, Material reward (behaviour); 10.3, Non-specific reward; 10.4, Social reward, 10.9, Self-reward; 10.10, Reward (outcome) | Identify an activity that the person values and inform them that this will happen if and only if they attend for health screening |
| 10.3 | Non-specific reward (behaviour) | Arrange delivery of a reward if and only if there has been effort and/or progress in performing the behaviour (includes ‘Positive reinforcement’) Note: if reward is material, code 10.2, Material reward (behaviour), if social, code 10.4, Social reward, and not 10.3, Non-specific reward; if reward is for outcome code 10.10, Reward (outcome). If informed of reward in advance of rewarded behaviour, also code one of: 10.1, Material incentive (behaviour); 10.5, Social incentive; 10.6, Non-specific incentive; 10.7, Self-incentive; 10.8, Incentive (outcome) | Identify something (e.g. an activity such as a visit to the cinema) that the person values and arrange for this to be delivered if and only if they attend for health screening |
| 7.1 | Prompts/cues | Introduce or define environmental or social stimulus with the purpose of prompting or cueing the behaviour. The prompt or cue would normally occur at the time or place of performance Note: when a stimulus is linked to a specific action in an if-then plan including one or more of frequency, duration or intensity also code 1.4, Action planning | Put a sticker on the bathroom mirror to remind people to brush their teeth |
| 12.1 | Restructuring the physical environment | Change, or advise to change the physical environment in order to facilitate performance of the wanted behaviour or create barriers to the unwanted behaviour (other than prompts/cues, rewards and punishments) Note: this may also involve 12.3, Avoidance/reducing exposure to cues for the behaviour; if restructuring of the social environment code 12.2, Restructuring the social environment; if only adding objects to the environment, code 12.5, Adding objects to the environment | Advise to keep biscuits and snacks in a cupboard that is inconvenient to get to  Arrange to move vending machine out of the school |
| 12.2 | Restructuring the social environment | Change, or advise to change the social environment in order to facilitate performance of the wanted behaviour or create barriers to the unwanted behaviour (other than prompts/cues, rewards and punishments) Note: this may also involve 12.3, Avoidance/reducing exposure to cues for the behaviour; if also restructuring of the physical environment also code 12.1, Restructuring the physical environment | Advise to minimise time spent with friends who drink heavily to reduce alcohol consumption |
| 2.3 | Self-monitoring of behaviour | Establish a method for the person to monitor and record their behaviour(s) as part of a behaviour change strategy Note: if monitoring is part of a data collection procedure rather than a strategy aimed at changing behaviour, do not code; if monitoring of outcome of behaviour, code 2.4, Self-monitoring of outcome(s) of behaviour; if monitoring is by someone else (without feedback), code 2.1, Monitoring of behaviour by others without feedback | Ask the person to record daily, in a diary, whether they have brushed their teeth for at least two minutes before going to bed  Give patient a pedometer and a form for recording daily total number of steps |
| 2.4 | Self-monitoring of outcome(s) of behaviour | Establish a method for the person to monitor and record the outcome(s) of their behaviour as part of a behaviour change strategy Note: if monitoring is part of a data collection procedure rather than a strategy aimed at changing behaviour, do not code ; if monitoring behaviour, code 2.3, Self-monitoring of behaviour; if monitoring is by someone else (without feedback), code 2.5, Monitoring outcome(s) of behaviour by others without feedback | Ask the person to weigh themselves at the end of each day, over a two week period, and record their daily weight on a graph to increase exercise behaviours |
| 15.4 | Self-talk | Prompt positive self-talk (aloud or silently) before and during the behaviour | Prompt the person to tell themselves that a walk will be energising |
| 3.1 | Social support (unspecified) | Advise on, arrange or provide social support (e.g. from friends, relatives, colleagues,’ buddies’ or staff) or non-contingent praise or reward for performance of the behaviour. It includes encouragement and counselling, but only when it is directed at the behaviour Note: attending a group class and/or mention of ‘follow-up’ does not necessarily apply this BCT, support must be explicitly mentioned; if practical, code 3.2, Social support (practical); if emotional, code 3.3, Social support (emotional) (includes ‘Motivational interviewing’ and ‘Cognitive Behavioural Therapy’) | Advise the person to call a ‘buddy’ when they experience an urge to smoke  Arrange for a housemate to encourage continuation with the behaviour change programme  Give information about a self-help group that offers support for the behaviour |
| 13.4 | Valued self-identity | Advise the person to write or complete rating scales about a cherished value or personal strength as a means of affirming the person’s identity as part of a behaviour change strategy (includes ‘Self-affirmation’ | Advise the person to write about their personal strengths before they receive a message advocating the behaviour change |
| 15.1 | Verbal persuasion about capability | Tell the person that they can successfully perform the wanted behaviour, arguing against self-doubts and asserting that they can and will succeed | Tell the person that they can successfully increase their physical activity, despite their recent heart attack |

*Note.* Adapted from Michie S., Richardson M., Johnston M., Abraham C., Francis J., Hardeman W., Eccles M., Cane J., and Wood C. (2013). The behavior change technique taxonomy (v1) of 93 hierarchically clustered techniques: building an international consensus for the reporting of behavior change interventions. Ann Behav Med, doi: 10.1007/s12160-013-9486-6).
