# Supplementary material for: Developing the PEAK mood, mind, and marks program to support university students’ mental and cognitive health through physical exercise: a qualitative study using the Behaviour Change Wheel
Source: BMC Public Health. 2024 Jul 23;24:1959. doi: 10.1186/s12889-024-19385-x (PMC11265317; doi:10.1186/s12889-024-19385-x)
Supplement: Supplementary file 4 — Supplementary Material 4 [file 12889_2024_19385_MOESM4_ESM.docx]

**Additional File 1.**

The Behaviour Change Wheel Intervention Types, Definitions and Examples

| **Intervention types** | **Definition** | **Example of intervention type** |
| --- | --- | --- |
| Education | Increasing knowledge or understanding | Providing information to promote healthy eating |
| Persuasion | Using communication to induce positive or negative feelings or stimulate action | Using imagery to motivate increases in physical activity |
| Incentivisation | Creating an expectation of reward | Using prize draws to induce attempts to stop smoking |
| Coercion | Creating an expectation of punishment or cost | Raising the financial cost to reduce excessive alcohol consumption |
| Training | Imparting skills | Advanced driver training to increase safe driving |
| Restriction | Using rules to reduce the opportunity to engage in the target behaviour (or to increase the target behaviour by reducing the opportunity to engage in competing behaviours) | Prohibiting sales of solvents to people under 18 to reduce use for intoxication |
| Environmental restructuring | Changing the physical or social context | Providing on-screen prompts for GPs to ask about smoking behaviour |
| Modelling | Providing an example for people to aspire to or imitate | Using TV drama scenes involving safe-sex practices to increase condom use |
| Enablement | Increasing means/reducing barriers to increase capability (beyond education and training) or opportunity (beyond environmental restructuring) | Behavioural support for smoking cessation, medication for cognitive deficits, surgery to reduce obesity, prostheses to promote physical activity |

Note. Reproduced from Michie S, Atkins L, West R. (2014) The Behaviour Change Wheel: A Guide to Designing Interventions. London: Silverback Publishing. [www.behaviourchangewheel.com](http://www.behaviourchangewheel.com).
