# Supplementary material for: Developing the PEAK mood, mind, and marks program to support university students’ mental and cognitive health through physical exercise: a qualitative study using the Behaviour Change Wheel
Source: BMC Public Health. 2024 Jul 23;24:1959. doi: 10.1186/s12889-024-19385-x (PMC11265317; doi:10.1186/s12889-024-19385-x)
Supplement: Supplementary file 5 — Supplementary Material 5 [file 12889_2024_19385_MOESM5_ESM.docx]

**Additional File 2.**

Student Focus Group Interview Schedule

**Introduction**

- Introduce PEAK, work to date, ultimate aims
- Describe purpose of focus groups generally

**Mental, cognitive and brain health impact of exercise**

- Show students’ video created by young adults: “*Exercising smart: how being active can help you perform better in your studies*”: <https://vimeo.com/ymcavictoria/download/535721809/3ed49f4683>

1. What are your reactions to the video that explained some of the mental, cognitive and brain health benefits of physical exercise?
2. To what extent has exercise had an impact on your mental health and cognition?

**Appetite for a program to support students’ mental and cognitive health**

1. How would you describe the mental and cognitive health status of university students?
2. To what extent is there a need for a university program to improve students’ mental and cognitive health?

**Barriers and facilitators to physical exercise**

1. What hinders and helps and helps your engagement with physical exercise?
   1. Prompt: Capability (knowledge / skills)
   2. Prompt: Opportunity (physical / social)
   3. Prompt: Motivation (reflective / automatic)

**Co-design**

1. If you were to design PEAK, what methods could be used to deliver the program?
2. If you were to design PEAK, what strategies would increase the adoption of the program?
   1. Prompt: What program outcomes are you interested in tracking (i.e. mental, exercise achievement, cognition) and what kind / frequency of feedback would you like?
   2. Prompt: What have/haven’t you found engaging in other physical exercise programs?

**Conclusion**

Are there any other comments you would like to make about PEAK, or topics we have discussed today?
