# Supplementary material for: Developing the PEAK mood, mind, and marks program to support university students’ mental and cognitive health through physical exercise: a qualitative study using the Behaviour Change Wheel
Source: BMC Public Health. 2024 Jul 23;24:1959. doi: 10.1186/s12889-024-19385-x (PMC11265317; doi:10.1186/s12889-024-19385-x)
Supplement: Supplementary file 6 — Supplementary Material 6 [file 12889_2024_19385_MOESM6_ESM.docx]

**Additional File 5.**

University Students’ Barriers and Facilitators to Exercise and the Anticipated Adoption of PEAK

| **COM-B category** | **Theme/ belief statement** | **Sub-theme/belief statement** | **Barrier/facilitator/mixed** | | **Example quote(s)** |
| --- | --- | --- | --- | --- | --- |
| CAPABILITY | | | | | |
| Psy C | My knowledge about exercise and how it is beneficial is variable | Learning how exercise impacts mental, cognitive and brain health is helpful | | Facilitator | *“The video [about how exercise impacts mental, cognitive and brain health] was very educative. I didn't actually know - I mean, I knew that exercise is healthy and helpful. But I thought it's okay not to exercise during the exam times, because you don't have time. You're too stressed. You cannot manage everything properly. Yeah, video was really great. Now I know that I have to exercise to help myself, because I didn't know how to relieve my stress during the exam period or other stressful times.”* FG 2, Female  *“I think this [video] was an eye opener. I need to do something physical, like physical exercise, during exam to help with stress, because I'm the person who will literally do nothing during exams, just study.”* FG 2, Female  *“I found that the video [about how exercise impacts mental, cognitive and brain health] was very informative as well…when you first just do exercise, oh, I'm just getting stronger or I'm feeling a bit happier, but you don't really think about the overall cumulative effects of it in general.”* FG 2, Male |
|  |  | I lack knowledge about how to exercise and use gym equipment | | Barrier | *“I don't really know how to use the gym.”* FG 4, Female  *“When I started going to the gym… I felt like I didn't know what anything did, and I would want to learn everything.”* FG 4, Female |
|  |  | I lack knowledge about how to manage the physical consequences (e.g. muscle soreness) and risks (e.g. injury) of exercise | | Barrier | *““Yeah. I would say something that would hinder exercise engagement, though, would be DOMS [Delayed Onset Muscle Soreness] or nervous system fatigue. When your muscles are very sore…”* FG 2, Male  *“Not many people know what soreness is okay to go to the gym or not. Even for me sometimes, I'm scared of snapping my tendons or whatever, right.”* FG 2, Male |
|  | I lack time management skills to fit exercise into my daily life | | | Barrier | *“Time is a big [barrier], being able to manage time.”* FG 1, Male  *“I mean for me I want to [exercise] I just find it difficult to schedule it into my timetable”* FG 3, Male |
|  | Tracking and receiving feedback on the progress and impact of my exercise is helpful/unhelpful | Tracking and receiving feedback on my exercise progress is helpful | | Facilitator | *“I used Apple before, but you have the step count like you said and it's kind of encouraging too. It’s a tiny thing like in the notification bar, the amount of steps you've done, but it's also very encouraging to see it next to like oh, this is the number of steps you have remaining to reach your goal. So it's always a very nice format and it's always encouraging to see that, so it helped me to walk more.”* FG 1, Female |
|  |  | Tracking and receiving feedback on the impact of exercise on my mental health and cognition is helpful/unhelpful | | Mixed | Barrier  ***“****I actually don't think [tracking mental health] is a huge factor……because if you do feel well, you feel well right? Whether you track it or not, I mean you'll just intrinsically feel well, so… I don't think that just recording it would increase it or decrease [my exercise], personally for me,”* FG 1, Female  *“I feel like logging [mental health change or thinking skill change] would be a bit [over the top] and a bit tedious.”* FG 2, Male  Facilitator  *“I think there's a lot to be said about recognising patterns in how you're feeling and if you recognise it, that'll sort of classically condition you to associate the two. That's just my perspective I guess.”* FG 1, Female  *“If [students are] seeing the benefits. If they're seeing emotional…benefits that would be probably enough to get them to continue [to exercise once the extrinsic incentive stopped].”* FG 4, Female  *“Yeah, [tracking and receiving feedback on thinking skills] would be nice actually. I think with the thinking skills and things, I think that would be really good as like a weekly summary”* FG 1, Male  *“Sometimes, especially when you do exercise for a continuous period, you start to notice results, but you forget what you were like before, and it's only until you see photos or you have measurements, or you do a certain task, that you're like, hold the phone. Wait, this has actually had a positive impact. I think maybe helping people identify a goal at the start and tracking it, that would definitely incentivise people to - at least me, personally, to keep going.”* FG 4, Female |
| Phys C | None identified | | | - | - |
| OPPORTUNITY | | | | | |
| Phys O | I don’t have enough time to exercise | | | Barrier | ***“****Having a big and busy day and you might not find time to get exercise in.”* FG 1, Female  *“I feel like it’s because we’re so time poor.  There’s always something else you can be doing.  I feel like with uni you’re always behind.”* FG 3, Female  “ I’d feel… too busy that I can’t exercise” FG 3, Male |
|  | I need [a range of] accessible exercise options that can flexibly suit my location, the weather, and exercise preferences | | | Facilitator | *“...making exercise accessible or whatever would be I think – I know that would appeal to me...”* FG 3, Female  “*I think [exercise] at home also helps accessibility a lot, because one of the reasons I stopped going to the gym was because I don't drive, and I don't have a car.  Going to the gym was like, it would take up my entire morning or afternoon, whenever I did it…It became very inaccessible.”* FG 4, Female  *“some people have bad home environment, so they can't work out at home. I went through that as well, so going to the gym was the actual place to go to exercise.”* FG 4, Female  *“One other thing might be like the accessibility to be able to do things maybe away from campus as well, so having some sort of online [program]”* FG 1, Male  *“Where possible, having outdoor things is great.  But then being able to have that back up and use indoor facilities in case of rain”* FG 1, Male  *“Having different types of exercise was really beneficial to our motivational levels…Like different types of exercise, so you can do strength based, high impact, just going for a run as a group and yeah, just different types of exercise you can do. Maybe you have yoga as well, just a recovery session with a bunch of other people. ”* FG 1, Male  *“Yeah, I think… a website system there which provides so much options like time we can choose the time and we can choose the type of the exercise.”*  FG 3, Male |
|  | Beginners need introductory exercise options to build confidence and learn basic exercise skills | | | Facilitator | *“…it would be better to have just some introductory classes…like learn how to exercise.”* FG 4, Female  *“I think it would be a good thing to always wrap the whole, whatever the duration of PEAK is, to always have that one set super-beginner class that's literally just teaching the skills. Everyone at least, at one point, would maybe have gone in that class, but then eventually gone to one of the other things that's offered… There should always be a skills and - which is a very basic, maybe for somebody who's never seen exercise before....”* FG 4, Female |
|  | I need exercise options that don’t need equipment | | | Facilitator | *“I think also including workouts that are – they use minimal equipment as well, because sometimes people get a little scared by…the amount of things you need to do the workout.”* FG 1, Female  *“So having a set of workouts that…don't require a lot of things, or just require things at home. That would be good.”* FG 1, Female  *“I don't really like using gym equipment”* FG 4, Female |
|  | Prompts to exercise are helpful | | | Facilitator | *“Input all the strategy initially into, say, a PEAK app or whatever it was and then it reminds you at that time, hey, by the way, you said that you wanted to exercise this time... A prompt from the phone that is set by you...”* FG 2, Male  *“I've been setting an alarm for every half an hour or an hour or something, and I'll just do 100 star jumps, and it's like a little restart.”* FG 4, Female |
|  | I am motivated/demotivated if I must pay money to exercise | | | Mixed | Barrier  *“I think it would be better for PEAK to be free.”* FG 4, Female  *“For me, I wouldn't pay anything at all [for PEAK].”* FG 4, Male  Facilitator  *“I also find with costs with gym memberships, it's almost like when you pay that's an obligation to attend the gym, so if it's a free program I just don't really – like there's no motivation behind it.”* FG 1, Female |
|  | I find it helpful/unhelpful to use existing university digital platforms to deliver the program, i.e. Moodle | | | Mixed | Barrier  *“I doubt people would actively do something on Moodle.”* FG 2, Male  *“I feel like most of the stuff that goes on with Moodle I ignore it… because on my Moodle I keep my units and I keep the general forum for my faculty and whatever.  There are other things like digital wellbeing or stuff like that which I completely ignore…If [the exercise program] were to go up on my Moodle it would have to be really advertised well and designed well.”* FG 3, Male  Facilitator  *“Another way where you could get all the students and - you know on your Moodle page you've got different tiles for units? If you could have a tile...with PEAK and that's - every student could see that, click on it and do whatever.”* FG 2, Male  *“Yeah, that’s why I don’t hate the Moodle idea if it was pushed through your faculty also kind of advertised maybe through social media.  If you saw an advertisement for it if it was done in the right way, I reckon it could be successful through Moodle.”* FG 3, Female |
| Soc O | Exercising with others is beneficial | Exercising with others increases motivation | | Facilitator | *“having people around to help you out with motivation.”* FG 1, Female  *“Because when you go to the gym, everyone's actually putting in the work. You feel motivated and stuff. But at home you have to find self-motivation. You have to put it in yourself.”* FG 2, Male  *“I find with my friends we workout so much better together, so PEAK could have something like - cool, you could have an individual program if you want. But you could also maybe merge two or three of your closest friends to actually work together… I think - if I'm paired with my friends, the people I've been with, we're more likely to push each other...”* FG 2, Male |
|  |  | Exercising with others helps keep me accountable | | Facilitator | *“… if I've made a commitment to say go to the gym on this day, with this person, I can't back out of it.”* FG 1, Female  *“I think group setting, group accountability [exercising] with other people….”* FG 4, Female |
|  |  | Exercising with others is enjoyable | | Facilitator | *“it's fun [exercising] with friends who you know. I think that's one motivator for me is like just doing it with people is fun...”* FG 1, Female |
|  |  | I enjoy socialising and making new friends through exercise | | Facilitator | *“being able to meet new people at group sessions is also great, especially if the program's going to be aimed undergrad students, because we want to meet people around the same age. Because it's so hard to make friends at uni anyway, so yeah.”* FG 1, Female  *“...if it's more of a comfortable community environment where they like going anyway, and it's also got a social factor to it as well, people don't feel like they're there for exercise, sometimes. It's like, you're just here regular once a week, you're seeing people and you just happen to be exercising. I think that's when the feel good can happen after, because you actually end up feeling good from the socialisation.”* FG 4, Female |
|  | Encouragement from others to exercise helps keep me accountable | | | Facilitator | *“My boyfriend is really good in helping me with [exercise]... he’s just like reminding me I should do some exercise today, okay, yeah, I’ll do it.”* FG 3, Female |
|  | I want options that can flexibly suit my preference to exercise alone or with others | | | Facilitator | *“Even during the one week, you might decide you're in more of a social mood. Sometimes, I can't stand being in social settings. Sometimes, I'd want to go to the class, when I'm feeling maybe a bit lower, and I'd need that push. But then when I'm in my super introverted kind of mood, I'd happily do a self-workout. If the option is there to do it. It would be bad if we were just forced to just do group classes only, and you're not a group person. Some people thrive working out by themselves. Yeah, that would be a big thing, the choice.”* FG 4, Female |
|  | I respond well to relatable exercise role models and champions to support the program | | | Facilitator | *“So it's nice to see maybe someone from MSA [Monash Student Association] or something like that who is more actively involved in student… life to talk about PEAK rather than someone higher up, because we never really know them or see them, so yeah.”* FG 1, Female  *“Sometimes we need to have a role model. Actually we have seen a certain person who has transformed before exercise and after exercise.  We may see it or when ourselves do start it and try it and see ourselves some transformation and we will have more aware – more willing to keep it.”* FG 3, Male |
|  | Being part of a supportive exercise community is helpful | | | Facilitator | *“Even though the actual people who were there, these people who were working super hard, they would come up to me on my first one, at the end of the workout, you did really good today.  Oh, I love this place. That's why I came back. That's the only reason.”* FG 4, Female  *“I think that [supportive community] is what really has drawn me into any fitness thing before, is everyone's so friendly and non-judgemental here.”* FG 4, Female |
| MOTIVATION | | | | | |
| Auto M | I feel too tired to exercise | | | Barrier | *“At the end of the day you might have time [to exercise], but then you're exhausted, or you just can't think about that right now. You just want to relax.”* FG 1, Female  *“I genuinely like going to the gym. I like before I go there, and then just halfway through, I'm just like, no, I can't do this. I'm capable, I just can't, my brain just says, no, you're too tired, this is too much, you've just got to go home.”* FG 4, Female  *“A bit like with maintaining it it would be hard because there are points in the semester where you’re like, I’m just so exhausted, I would do anything to just sleep let alone like do exercise.”* FG 3, Female |
|  | I don’t feel like exercising when I’m experiencing poor mental health | | | Barrier | *“I think your mental state also would play a big role so if you're sad or depressed as we discussed... If I'm sad, I would not want to exercise. I'm just sad and stuff.”* FG 2, Female  *“But I'm not used - I don't exercise regularly. I just exercise when I feel like it, because I'm a very emotional person. My mental state, my mood would really determine my abilities of doing anything.”* FG 2, Female  *“For a mental health situation sometimes our mental health is not good enough and we may just skip the exercise.”* FG 3, Male  *“Yeah, I feel like because we're so stressed about the exams coming up and we start to neglect about everything else. Because we start to stress more, we don't think about, oh, maybe if we had only about an hour exercise, it would actually help us.,.”* FG 2, Male |
|  | I feel self-conscious exercising in front of others | | | Barrier | *“…I worked out with some guy friends…I didn't really enjoy it, because they used to comment, oh, your thighs are thicker than mine and stuff like that. I would feel insecure working around with people that I know.”* FG 2, Female  “*When I started going to the gym, it was really intimidating… you're so scared that you're going to do something, and people are going to be like, what is this girl trying to do?*” FG 4, Female |
|  | I want exercise to be enjoyable | | | Facilitator | *“...[exercise] needs to be fun as well.”* FG 4, Male  *“I think, what I remember positively about PE was the fun games that we would play, like dodgeball and stuff like that.”* FG 4, Female |
|  | Having a routine helps me exercise | Having an exercise routine is helpful | | Facilitator | *“…building up momentum is such an important thing when it comes to exercising I reckon.”* FG 1, Female  *“If you have an [exercise] routine going, it helps you flow.”* FG 2, Male |
|  |  | Having a healthy sleep routine helps my exercise | | Mixed | Barrier  *“I think the rest of the time when you have uni, you have a very crazy sleep schedule. I sometimes find myself sleeping at 2am, 3am, which means I wake up really late as well. So it's a late start to the day and it's really hard to find motivation or the energy to do a workout, so yeah, that's one of the things I would say.”* FG 1, Female  Facilitator  *“…if I had like nine, 10 hours [of sleep]... I'd be like, okay, I need to be active.”* FG 2, Female |
|  |  | Having a healthy diet routine helps my exercise | | Facilitator | *“I also find that my eating habits dictate how I feel in terms of going into a workout, so if I've been eating healthily around a certain time, I find the workouts that I do in that time are always more effective and yeah, I don't know. It's almost like the momentum's built into it as well, like if I keep up healthy meals and also exercising it's almost like an ego boost”* FG 1, Female  *“Another thing we discussed was diet. Say if you have a routine with diet, you'll be eating healthy, you're more likely to put in the work [to exercise] and stuff.”* FG 2, Male 3 |
|  | I respond well to incentives to exercise | | | Facilitator | “*Yeah and then also with a goal, having a reward at the end. That personally [laughs] motivates me, if I know that I've done my exercise for the month or the week or whatever, yeah and done what I planned to do, then I like to reward myself for that.”* FG 1, Female  *“We love extrinsic motivators [to exercise]”* FG 1, Male  “*…You just have to maybe give some reward to students...because we are really motivated by rewards. Anything related to...vouchers, money, food, we'll love it”* FG 2, Female |
| Ref M | I don’t prioritise exercise (because of my study) |  | | Barrier | *“during assignments and exams, it becomes less of a priority because you're trying to have as much time to do your studies then too…it’s really hard to balance it with the amount of assignments we have”* FG 1, Female  *“I guess I also find that for uni, the workload is a lot higher compared to high school. So yeah, in a way that kind of hinders me because I feel like I've got to spend more time studying, or working on assignments and that takes away from the time I could be spending on being outside, engaging in physical activities.”* FG 1, Female  “*But problem with gym was that lack of motivation, because I always prioritise my studies and work over my fitness*.” FG 2, Female 3  *“I think I'm a person who stops doing everything during exams. I cancelled my gym membership in the exam month. I put it on hold. I don't go out.”* FG 2, Female |
|  | I find it helpful to set exercise goals |  | | Facilitator | *“… if we set a goal, it helps us to do [exercise] and I find that for myself as well. If I set a specific goal, then that's more likely that I'll do it as well, the sense of fulfilment that we would get from that as well helps.”* FG 1, Female  *“I think setting more achievable goals helps to start [exercise] and maybe even – because right now my goal is just like get moving and should really sound like achievable like I can go for a walk and do like 15-minute exercise like that’s achievable.”* FG 3, Female  *“I guess the fact that you can change those goals whenever you want, it's very up to you, you don't have to be pressured into, I need to do 30 minutes of exercise. I can do 10 minutes, start small, and then just increase along the way. Then looking back, oh, yes, I started with this, now I can do an hour a day, and that's very rewarding, I think.”* FG 4, Female  *“For me, what I've noticed is when I have a specific fitness goal that I want to reach, and I've created a plan to help myself reach that goal, that motivates me significantly more than if I'm just kind of like, I don't have a goal to achieve, or I do have a goal, but I don't know how to get there. I think helping people identify what goals they have and how to make a plan to reach that goal, would definitely be a huge help.”* FG 4, Female |
|  | Exercise has a positive/negative impact on my mental health and wellbeing | Exercise helps me feel less stressed | | Facilitator | “*[Exercise is] a good stress relief*” FG 2, Male  *“Less stress [when exercising]”* FG 3, Female  *“But then sometimes like in exam week or in the week leading up to exam week when you’re so stressed I will find myself going on walks or the occasional very infrequent run”* FG 3, Female |
|  |  | Exercise has a positive/negative impact on my mood | | Mixed | “Facilitator  *“[Exercise] makes you happy, like you feel better*” FG 3, Female  “*When I do exercise now, I just notice a huge upshoot in how good I feel. It's very, very drastic.”* FG 4, Female  Mixed  *“I find light exercise, in smaller increments, to be quite beneficial in improving my mood, but if I set out to do a real intense, strenuous activity, I find I just feel awful. Just exhausted and gross and sweaty and not happy.”* FG 4, Female |
|  |  | Exercise increases my self-esteem | | Facilitator | *“[Exercise] increases your self-esteem”* FG 2, Female |
|  |  | Exercise helps me to feel a sense of self-achievement | | Facilitator | ““*The feeling of accomplishing that hardened task would be a good feeling… for let's say team sports and tournaments and the like, when you win as a team or if you're doing for instance resistance training, then at the end of it, after smashing it out, [a very heavy weight or very light even]. It's like I was dead in the middle of it, but at the end I've done it. I feel really happy that I did the goal that I wanted to accomplish.”* FG 2, Male  *“I get often a sense of achievement [from exercise]”* FG 3, Male |
|  | Exercise has a positive/negative impact on my thinking skills | Exercise helps me to stay focused for longer | | Facilitator | “*I find I'm able to focus for longer.”* FG 1, Female  *“I feel…just being able to focus a bit more, rather than drifting the whole time”* FG 4, Female |
|  |  | Exercise helps me to focus on one task at a time | | Facilitator | “*when I have the time to do exercise and stuff…I can focus on one thing”* FG 1, Male  *“ I can focus more on one thing, instead of jumping between tasks”* FG 1, Female |
|  |  | It is difficult to think critically, and problem solve directly after engaging in vigorous exercise | | Barrier | *“…on these particular days, in the mornings, after my exercise, I just wouldn't be able to think as critically to solve all the problems I needed to deal with… For the few hours after the exercise”* FG 4, Male |
|  | The physical impact of exercise is positive/negative | Exercise has a positive/negative impact on my energy | | Mixed | Barrier  *“I feel like if I do work out I'm going to expend energy, energy which I might use to study…”* FG 1, Female  *“I get really sleepy during my workout, when I get home, I have to nap. I cannot do another task. If you tell me, get home, study, go get some errands, go home - I literally have to allocate time to sleep.”* FG 4, Female  Facilitator  *“Yes, exercise stops you being so lethargic and makes me feel physically healthier”* FG 4, Female  *“I notice, as soon as I start doing exercise, I just pretty instantly, just like that, [my energy] shoots way up.”* FG 4, Female |
|  |  | Exercise has a positive impact on my strength and fitness | | Facilitator | “*That's one reason why I started working out, because I wanted to be fitter”* FG 2, Female |
|  | Exercise has a positive impact on my appearance | | | Facilitator | “*You look better and you think you look better. Somehow after exercising, I see improvements. My face becomes slimmer right away.”* FG 2, Female  *“if you gained weight and you're not happy with your reflection, you're more likely to exercise, not because you like exercising but just because you have to get back in your previous shape just to feel good, to feel yourself again.”* FG 1, Female |

*Note.* Psy C, psychological capability; Phys C, physical capability; Phys O, physical opportunity; Soc O, social opportunity; Ref M, reflective motivation; Auto M, automatic motivation; FG, focus group
