# Supplementary material for: Developing the PEAK mood, mind, and marks program to support university students’ mental and cognitive health through physical exercise: a qualitative study using the Behaviour Change Wheel
Source: BMC Public Health. 2024 Jul 23;24:1959. doi: 10.1186/s12889-024-19385-x (PMC11265317; doi:10.1186/s12889-024-19385-x)
Supplement: Supplementary file 7 — Supplementary Material 7 [file 12889_2024_19385_MOESM7_ESM.docx]

**Additional File 4.**

The APEASE Criteria for Designing Behavioural Interventions

| **Criterion** | **Description** |
| --- | --- |
| Affordability | An intervention is affordable if within an acceptable budget it can be delivered to, or accessed by, all those for whom it would be relevant or of benefit. |
| Practicability | An intervention is practicable to the extent that it can be delivered as designed through the means intended to the target population. |
| Effectiveness and cost-effectiveness | Effectiveness refers to the effect size of the intervention in relation to the desired objectives in a real-world context. Cost-effectiveness refers to the ratio of effect to cost. If two interventions are equally effective, then the most cost-effective should be chosen. If one is more effective but less cost-effective than another, other issues such as affordability, come to the forefront of the decision-making process. |
| Acceptability | Acceptability refers to the extent to which an intervention is judged to be appropriate by relevant stakeholders (public, professional and political). Acceptability may differ for different stakeholders. |
| Side-effects/safety | An intervention may be effective and practicable but have unwanted side-effects or unintended consequences. These need to be considered when deciding whether to proceed. |
| Equity | An important consideration is the extent to which an intervention may reduce or increase the disparities in standard of living, wellbeing, or health between different sectors of society. |

Note. Adapted from Michie S, Atkins L, West R. (2014) The Behaviour Change Wheel: A Guide to Designing Interventions. London: Silverback Publishing. [www.behaviourchangewheel.com](http://www.behaviourchangewheel.com)
